# Recent advances in the synthesis of thienoindole analogs and their diverse applications

**DOI:** 10.1039/d1ra09233b

**Published:** 2022-05-25

**Authors:** Shubham Pandey, Simran Aggarwal, Ritu Choudhary, Satish K. Awasthi

**Affiliations:** Department of Chemistry, Chemical Biology Laboratory, University of Delhi 110007 Delhi India satishpna@gmail.com remarkableshubh@gmail.com aggsimran22@gmail.com ritu.choudhary0190@gmail.com

## Abstract

Thiophene-fused heterocyclic organosulfur systems, especially the thieno[3,2-*b*]indole moiety have attracted significant attention because they show a wide spectrum of biological activities such as antituberculosis, antitumor, antifungal, antibacterial, and human 5-HT5A receptor binding inhibition. Moreover, they also find applications in material chemistry and chemical engineering. Thus, due to their intriguing properties and applications, researchers are continually attempting to create more effective and environment-friendly methods for their preparation. In this review, we present a complete assessment of the current advances in the field of thieno[3,2-*b*]indole synthesis.

## Abstract 2

Synthesis of thienoindoles *via* easy and atom economical approaches followed by their potent applications.

## Graphical abstract

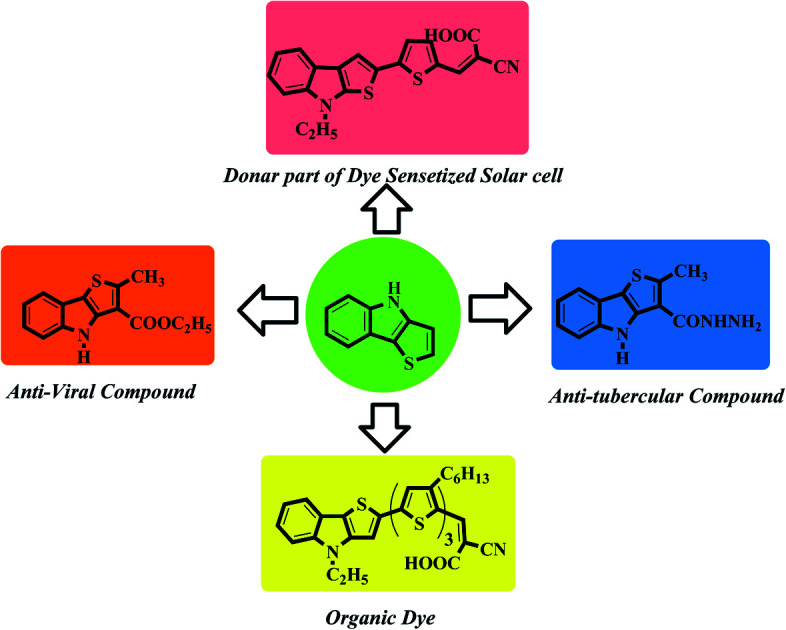

## 1 Introduction

Thiophene-fused heterocyclic organosulfur systems have piqued the interest of chemists around the world as they exhibit a diverse set of biological properties and are considered safe compounds for agricultural and pharmaceutical applications.^1^

**Fig. 1:**
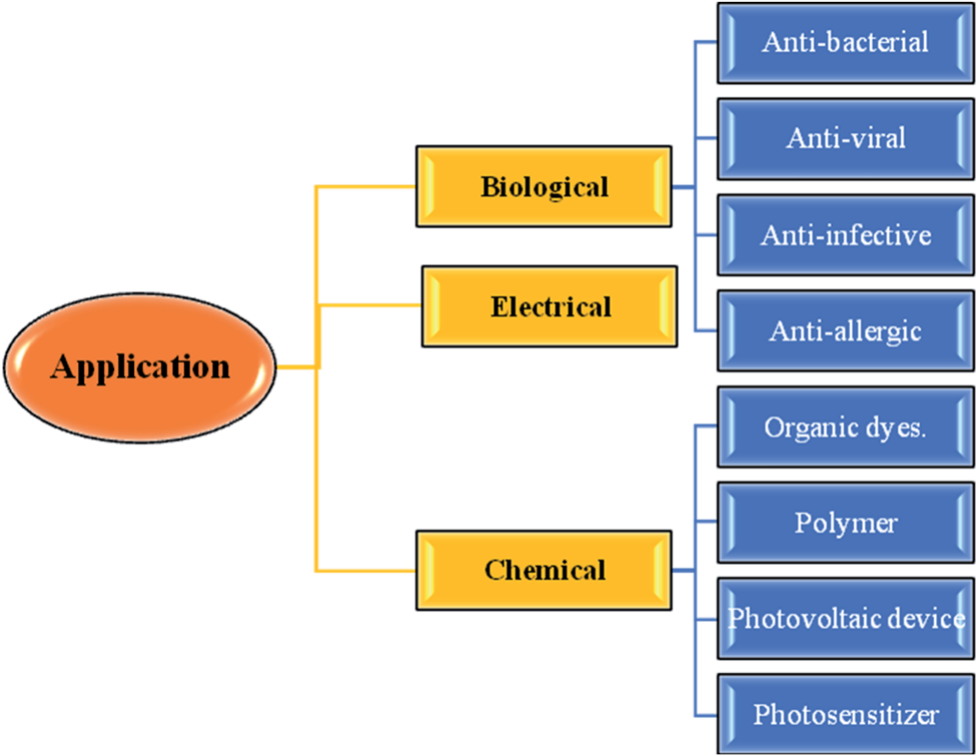
Applications of thienoindoles.

The thieno[3,2-*b*]indole moiety is specifically useful in the development of antituberculosis,^2^ human 5-HT5A receptor binding inhibition, antitumor,^3^ anti-infective, anti-osteoarthritis,^4^ antibacterial,^5^ and antifungal^6^ drugs and also potent in curing neurological diseases such as senile dementia and Parkinson's disease (Fig. 1).

**Fig. 2:**
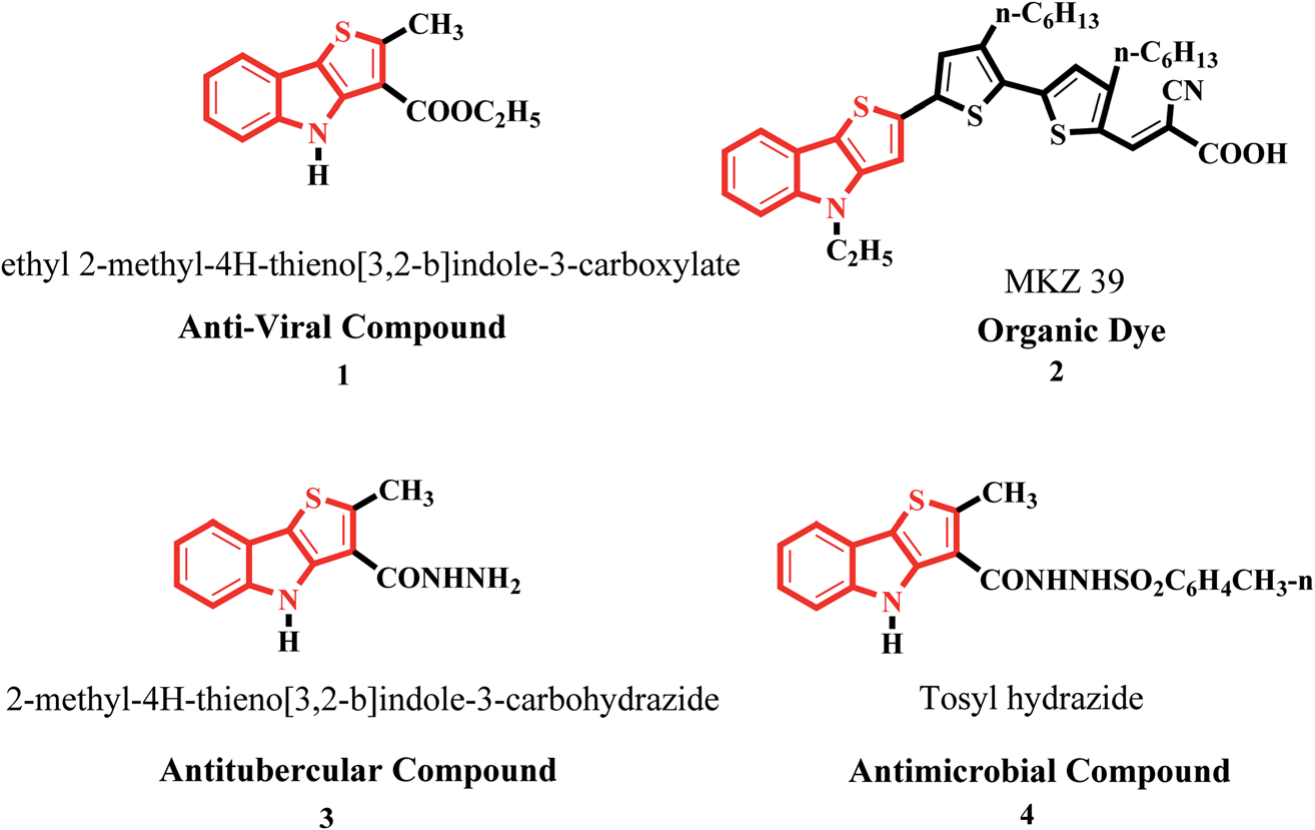
Substituted thieno[3,2-*b*]indole derivatives and their application.

Moreover, it is an important type of π-extended electron-rich system, which can be used for designing molecules for photosensitive and photovoltaic devices. In the past few years, it has also been widely used in designing and engineering fused molecules for organic electronic application, which basically works *via* the electron push–pull mechanism. This moiety is present in several functionalized organic dyes^7–9^ such as MKZ-39 and DPP-r-TI, which is an effective photosensitizer for photothermal and photodynamic therapies and polymers^10,11^ such as PTITBT, PTTICN, PTTIF, and PTTI (Fig. 2).

**Fig. 3:**
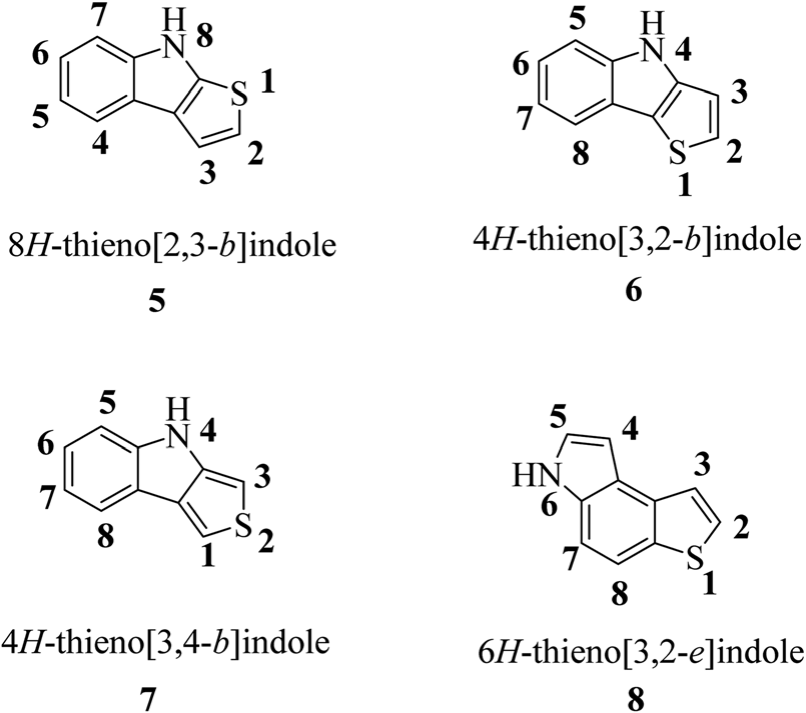
Types of thienoindoles.

According to the fusion of the thiophene ring on the indole ring, thienoindole can be categorized into different types. It is a tricyclic heterocyclic compound in which a thiophene ring is fused to an indole ring having one nitrogen atom. The fusion may occur in four different ways, *i.e.*, 8*H*-thieno[2,3-*b*]indole 5, 4*H*-thieno[3,2-*b*]indole 6, 4*H*-thieno[3,4-*b*]indole 7 and 6*H*-thieno[3,2-*e*]indole 8, resulting in four important types of thienoindoles (Fig. 3).

## 2 Synthesis of thienoindoles

**Scheme 1:**
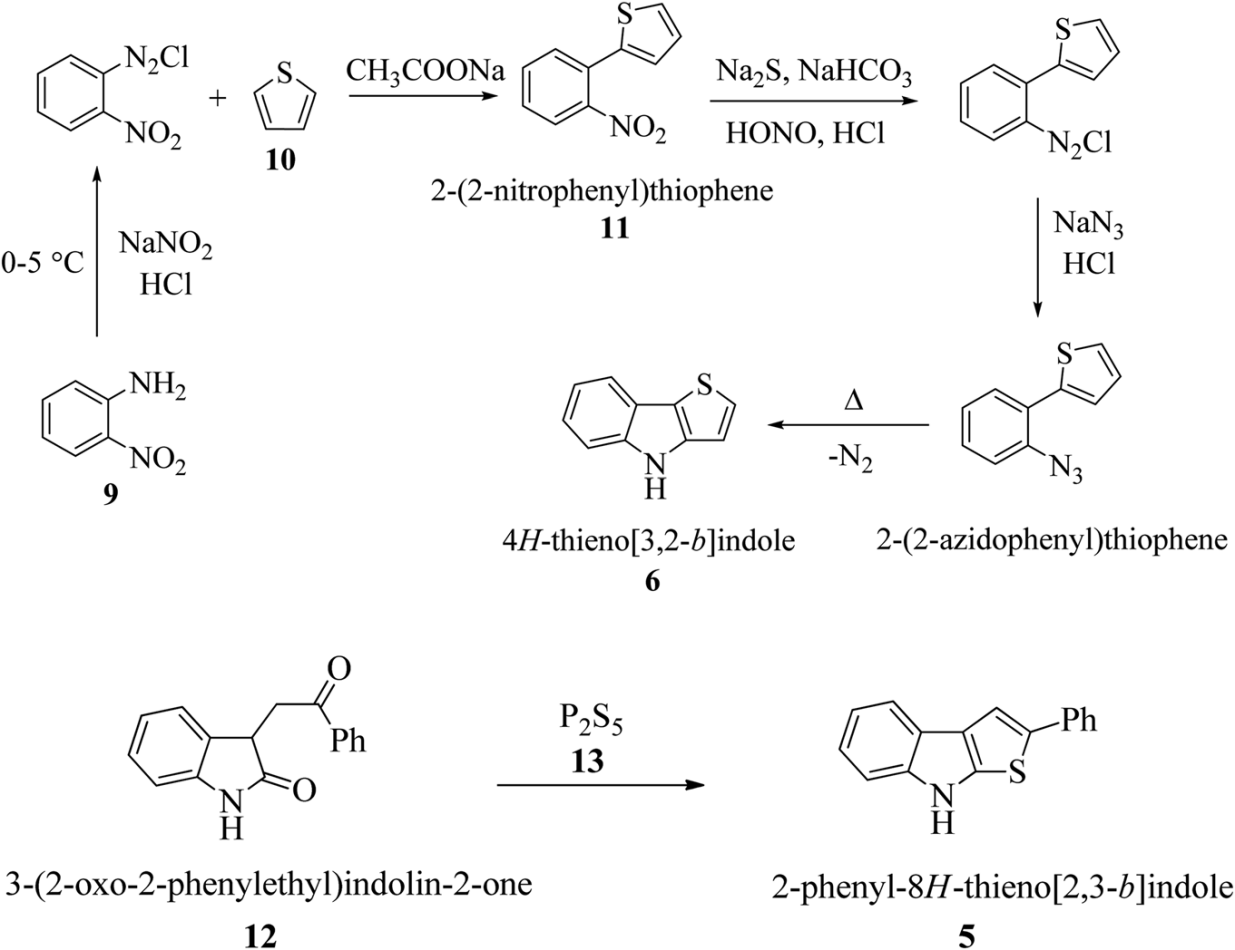
(a) First synthesis of thieno[3,2-*b*]indole *via* diazotization of *o*-nitroaniline and thiophene. (b) First synthesis of thieno[2,3-*b*]indole from 3-phenacyloxindole and phosphorous pentasulfide.

In the past few decades, thiophene-fused indoles have gradually been established as a novel class of valuable compounds having intriguing chemical activities and distinct biological activities. Plant growth regulators are critically important for crop production in high yield and enhanced quality, and therefore many plant growth-regulating chemicals have been synthesized to yield good seedlings by promoting root elongation. Nowadays, synthetic chemicals are used at various stages of rice plant development. A streptomycete strain identified as *Streptomyces albogriseolus* MJ286-76F7 produces a novel active chemical named thienodolin, an alkaloid having a thienoindole skeleton, which exhibits growth-promoting and growth-inhibiting activities in rice seedlings. In 1950, P. A. S. Smith and co-workers reported the first synthesis of 4*H*-thieno[3,2-*b*]indole 6 from the diazotization of *o*-nitroaniline 9 and thiophene 10*via* the formation of 2-(2-nitrophenyl)thiophene 11 (Scheme 1a). Later, in 1960, Kobayashi *et al.* synthesized thieno[2,3-*b*]indoles 5, starting from 3-(2-oxo-2-phenylethyl)indolin-2-one 12 and phosphorus pentasulfide 13 (Scheme 1b). In the early 90 s, Nakamura *et al.* structurally elucidated and Kanbe *et al.* isolated and characterized thienodolin by actively extracting thienodolin from a *Streptomyces albogriseolus* culture broth using ethyl acetate as the solvent followed by purification *via* preparative HPLC and silica gel column chromatography. It was found that when rice seedlings were treated with 1.2 × 10^−6^ to 1.2 × 10^−5^ M thienodolin, it exhibited growth-promoting activity, whereas 4.0 × 10^−5^ M thienodolin showed inhibitory activity in rice seedlings.

### 2.1 Synthesis of thieno[2,3-*b*]indole by radical cyclization

**Scheme 2:**
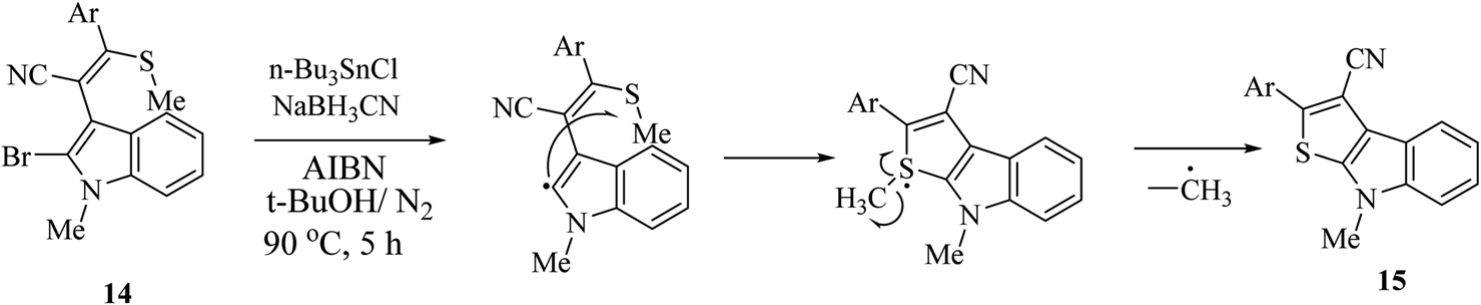
Radical cyclization approach for the synthesis of 15.

Singh *et al.*^12^ synthesized substituted thieno[2,3-*b*]indole 15 using the radical cyclization approach in 2011 (Scheme 2). However, although this is an effective approach for the synthesis of thieno[2,3-*b*]indole in high yield, the substrate, (*o*-bromoindolyl)acrylonitrile 14, used is synthesized in many steps *via* the base-induced condensation of (*o*-bromoindolyl)acrylonitrile with various aryl/heteroaryldithioesters. Also, the ^1^H NMR spectrum of the substrate showed that the substrate is produced as an inseparable mixture of (*E*)/(*Z*) isomers. Moreover, the push–pull nature of the double bond forced the substrate to undergo thermal (*Z*)/(*E*) isomerization.

Thieno[2,3-*b*]indole and its derivatives were also prepared *via* the nitrene-mediated Cadogan cyclization of 3-(*o*-nitrophenyl)thiophene, AlCl_3_-induced electrophilic recyclization of 2-(2-furyl)aryl isothiocyanates and oxidative cyclization of indolin-2-thiones. However, most of these methods are afflicted by drawbacks of multistep precursor synthesis, limited scope and generality. Accordingly, considering their applications in pharmaceuticals and materials science, more versatile and effective strategies for the synthesis of thiophene-fused heterocycles are needed.

### 2.2 Synthesis of 2-substituted thieno[2,3-*b*]indoles by Lawesson's reagent (LR)

Igrashev and co-workers reported the convenient, short and reliable synthesis of 2-substituted thieno[2,3-*b*]indoles from readily available reagents involving the two-step reaction-aldol-crotonic condensation of the starting materials and treatment of the intermediate with Lawesson's reagent. The reaction of the intermediate with LR takes place in two steps. Initially, the ethylidene double bond of indolin-2-ones undergoes reduction, and then Paal–Knorr cyclization occurs to give the tricyclic product.

**Scheme 3:**
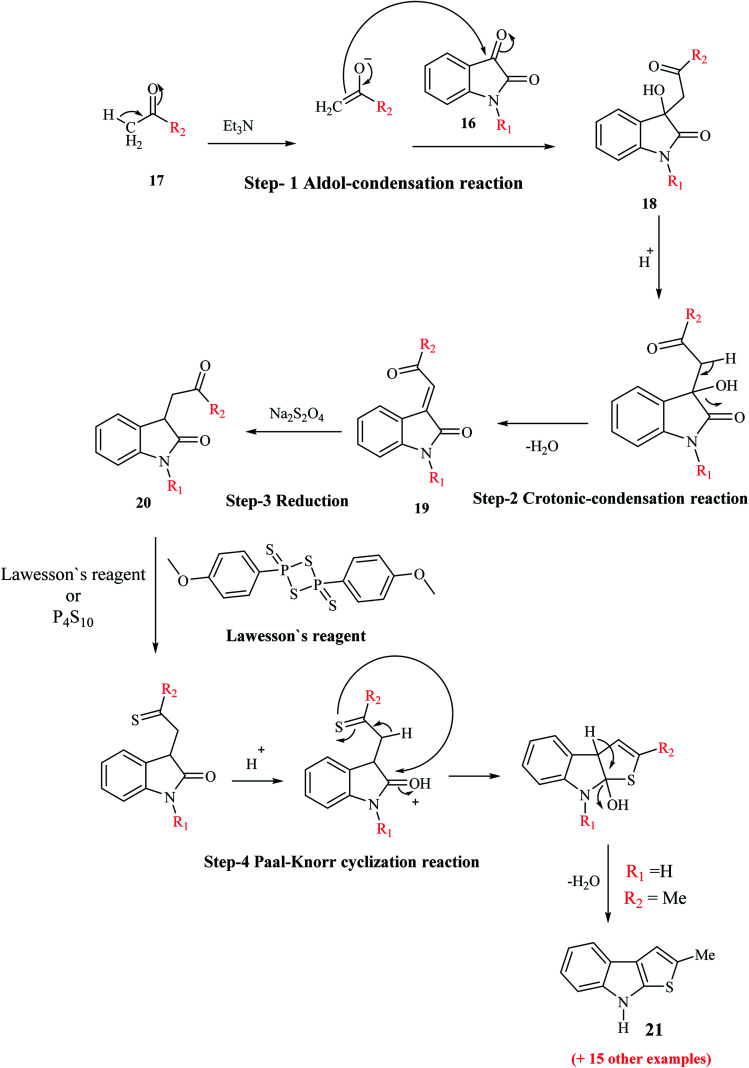
Synthesis of 2-substituted thieno[2,3-*b*]indole 21*via* thionation of indolin-2-ones 19.

When isatins 16 are treated with methyl ketones 17 in mild base, *e.g.*, secondary and tertiary amines, aldol-type adduct 18 is formed, which undergoes dehydration under acidic conditions to form the crotonic condensation product 3-(2-oxo-2-(hetero)arylethylidene)indolin-2-one 19. Compound 19 is more stable than compound 18 given that compound 19 is an unsaturated 1,4-diketone. The carbon–carbon double bond of 19 undergoes reduction in the presence of Na_2_S_2_O_4_,^13^ H_2_/Pd(C)^14^ or Me_3_P–H_2_O (ref. 15) (Scheme 3) to give indolin-2-one 20. Compound 20, which bears a 4-oxobutyramide fragment (1,4-dicarbonyl derivatives), undergoes Paal–Knorr reaction in the presence of thionation agents such as P_4_S_10_ or Lawesson's reagent and gets cyclized into thieno[2,3-*b*]indole 21.

Depending on the reaction conditions, the Paal–Knorr reaction produces pyrroles, furans or thiophenes from 1,4-diketones. Thiophene is obtained by using sulfurization agents such as phosphorus pentasulfide and Lawesson's reagent. LR is used as a thiation agent and is a powerful dehydrator, driving the reaction towards completion. This reagent has a four-membered ring of alternating phosphorus and sulfur atoms.

Although this synthetic strategy appears to be appropriate, it has little preparative interest given that thienoindoles are obtained in low yields. For example, 2-methyl-8*H*-thieno[2,3-*b*]indole was obtained in 15% yield *via* this four-step pathway using unsubstituted isatin and acetone.

**Scheme 4:**
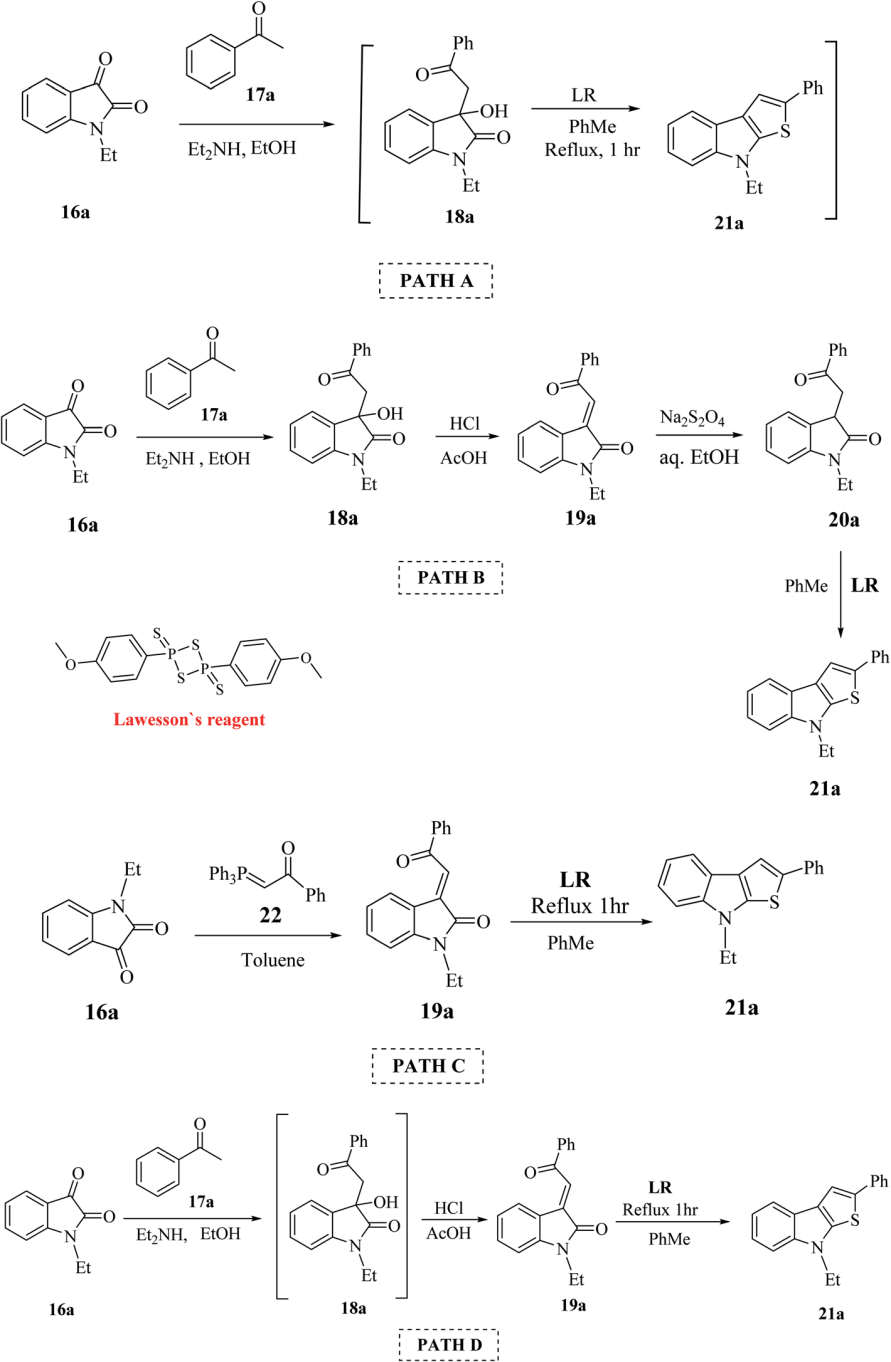
Various paths undertaken for enhancing the yield of thienoindoles.

This procedure was further modified using path A to path D to enhance the overall yield of the desired product. Thieno[2,3-*b*]indole 21a was prepared using 1-ethyl-isatin 16a and acetophenone 17a in the presence of base and ethanol *via* path A to path D (Scheme 4).

#### 2.2.1 Path A

Compound 18a was refluxed with LR in toluene for 1 h and the target compound 21a was obtained in very poor yield (10%).

#### 2.2.2 Path B

This is the conventional path to get 19a*via* the dehydration of aldol adduct 18a. Further, the reduction of 19a generates indolin-2-ones 20a, which undergoes cyclization using LR to give substituted thienoindole 21a (25%).

#### 2.2.3 Path C

This is a one-pot synthetic route, which involves the reaction of isatin 16a with (phenacylidene)triphenylphosphorane 22 to give intermediate 19a, which gets cyclized to give 54% of 21a. However, the limitation of this method is the use of phosphorane derivative 22 (formed by pre-functionalization of acetophenone 17a), which is very expensive.

#### 2.2.4 Path D

This pathway involves the refluxing of intermediate 19a with LR in toluene for 1 h, giving 21a in 57% yield.

Lawesson's reagent first acts as the source of H_2_S to reduce C=C in 19a, and then acts as the thiation agent to give 21a*via* Paal–Knorr reaction. Thus, the four-step procedure is reduced to a two-step procedure, leading to an overall good yield of the product. Hence, path D is the most effective, shortest and most robust method for the synthesis of thienoindoles. In some specific cases, path C is also used as an alternative synthetic route.

**Fig. 4:**
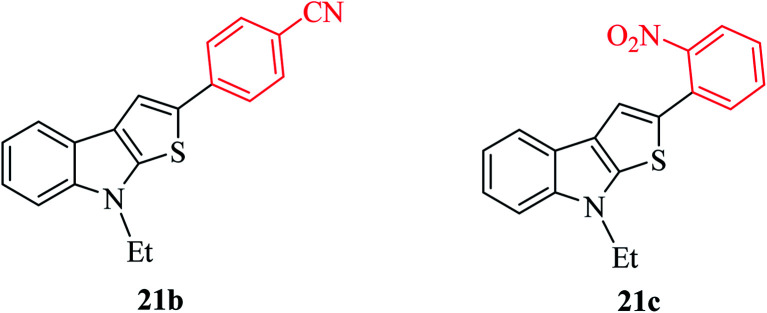
Thieno[2,3-*b*]indole containing electron-withdrawing groups.

Thieno[2,3-*b*]indoles containing electron-withdrawing groups such as 4-cyano or 2-nitro-phenyl substituent at the C-2 position were prepared in high yields of 92% and 82%, respectively (Fig. 4).

### 2.3 Synthesis of thienoindoles *via* Pd-catalyzed cross coupling reaction

#### 2.3.1 Pd-Catalyzed cross-coupling reactions

Palladium can transfer two electrons and form complexes in the 0 and +2 oxidation state. According to Pauling's scale, Pd has an electronegativity of 2.2, which leads to the formation of relatively stable and non-polar Pd–C bond. Thus, Pd is extensively used in synthesis. Due to the capacity of Pd to interact with non-polar bonds, a heteroatom a lone pair of electrons can easily undergo oxidative addition, transmetalation and reductive elimination. Heck, Negishi and Suzuki pioneered the work on Pd-catalyzed cross-coupling reactions and were awarded the Nobel prize in 2010.

#### 2.3.2 Suzuki–Miyaura cross-coupling reaction

The Suzuki–Miyaura reaction is a coupling reaction between aryl halides and organoborane reagents, including boranes, boronic acids and boronic esters. Organoboranes are non-toxic, air and moisture resistant and can be easily handled. The non-polar nature of the C–B bond (because of the relatively lower electronegativity of boron) makes it more stable than other metal–carbon bonds such as Li, Mg, Si, Al, Zr, Cu and Sn.

##### 2.3.2.1 Role of base and solvent

**Scheme 5:**
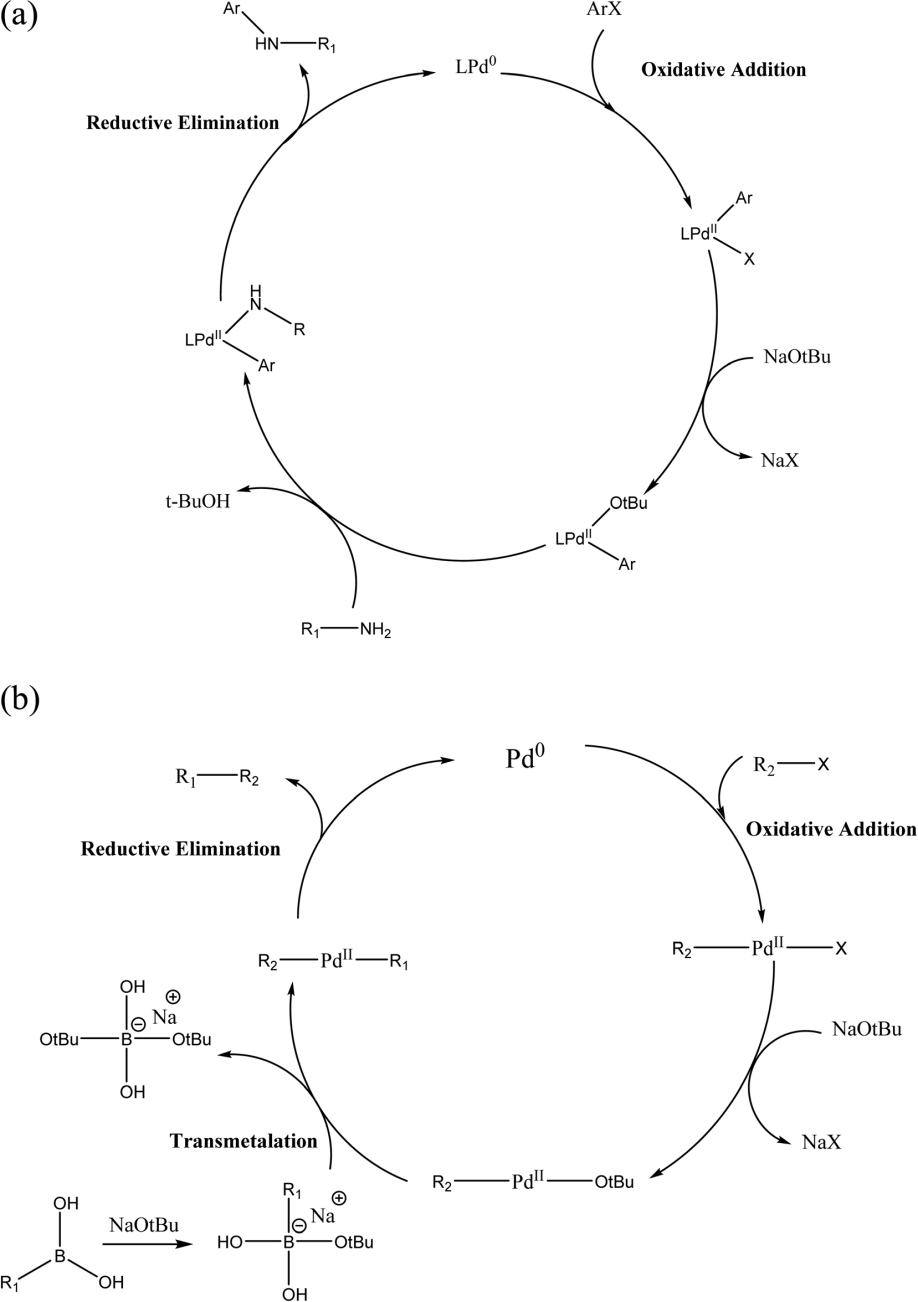
(a) Mechanism of Suzuki–Miyaura cross-coupling reaction; (b) Buchwald–Hartwig coupling reaction.

In most organoboron compounds, the C–B bond is extremely covalent and the complex does not undergo transmetalation easily. Thus, the notable and significant role of the base such as K_2_CO_3_, K_3_PO_4_, Na_2_CO_3_, NaOH and NaHCO_3_ in the Suzuki–Miyaura reaction is to activate the organoboron derivative by making a hypervalent, anionic boron-“ate” complex, which promptly undergoes transmetalation. In an alternative process, the base displaces the halide in the [PdXR^2^] complex to form the [Pd(O*t*Bu)R^2^] complex (Scheme 5).

The activity and selectivity of the Suzuki–Miyaura reaction are influenced by the solvent such as PhMe, DMF, 1,4-dioxane, benzene, THF and CH_3_CN. Moreover, a mixture of organic solvents and water can be utilized to increase the rate, selectivity and yield of the coupled product. In the current scenario of synthesis, a mixture of toluene and 1,4-dioxane is used as the organic solvent and water as the co-solvent.

#### 2.3.3 Buchwald–Hartwig amination reaction

A Pd catalyzed cross-coupling reaction between amines and aryl halides forms the C–N bond. In the case of P(*t*-Bu)_3_, a monodentate ligand, the active Pd[P(*t*-Bu)_3_] is formed. Imine is obtained as a by-product due to the β-hydride elimination reaction, which occurs when the H-atom is at the α-position to the N-atom.

Toluene and 1,4-dioxane are frequently used as solvents given that they have a high boiling point and can solubilize most organic compounds. Generally, strong bases such as NaO*t*Bu and KO*t*Bu in toluene are used to increase the reaction rate and product yield.

#### 2.3.4 Synthesis of thieno[3,2-*b*]indoles and thieno[3,4-*b*]indoles

**Scheme 6:**
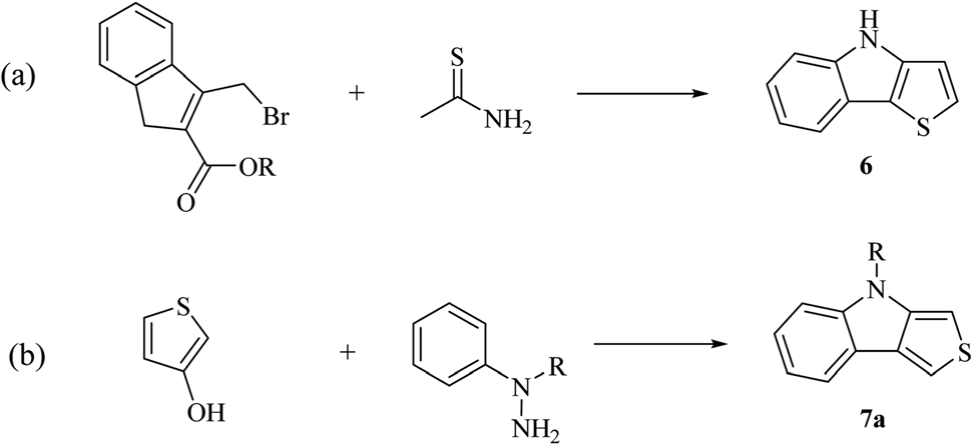
Classical synthetic approaches for synthesizing 6 and 7a.

Thieno[3,2-*b*]indoles 6 and thieno[3,4-*b*]indoles 7a were firstly synthesized in 1982 *via* two methods (Scheme 6), as follows:

(a) Reaction of substituted indole with thioamide.^16^

(b) Reaction of 3-hydroxy thiophene with hydrazine.^2^

However, these methods were tedious, inflexible and no improvement was reported until 2000. Later, the development of Pd cross-coupling chemistry facilitated the synthesis of thienoindoles and a palladium-catalyzed two step procedure has been developed, which involves a Suzuki reaction to form a C–C bond between benzene and thiophene, followed by ring closure. Ring closure reaction can be of three types, as follows: (a) nitrene insertion,^17^ (b) oxidative C–N coupling^18^ and (c) Cadogan cyclization.^19^

**Scheme 7:**
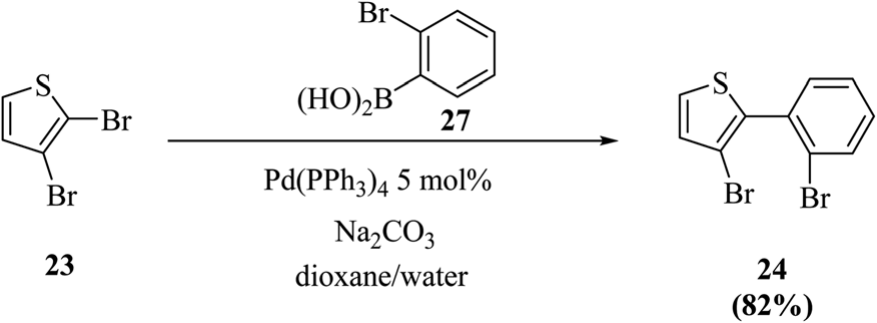
Site-selective Suzuki cross-coupling of 2,3-dibromothiophene.

Thieno[3,2-*b*]indoles were synthesized efficiently *via* the site-selective Suzuki–Miyaura coupling of 2,3-dibromothiophene with 2-bromophenylboronic acid,^20^ and subsequent two-fold palladium catalyzed C–N coupling (Buchwald–Hartwig reaction). In the first step, 2,3-dibromothiophene 23 is converted to 3-bromo-2-(2-bromophenyl)thiophene 24 in 82% yield (Scheme 7).

**Scheme 8:**
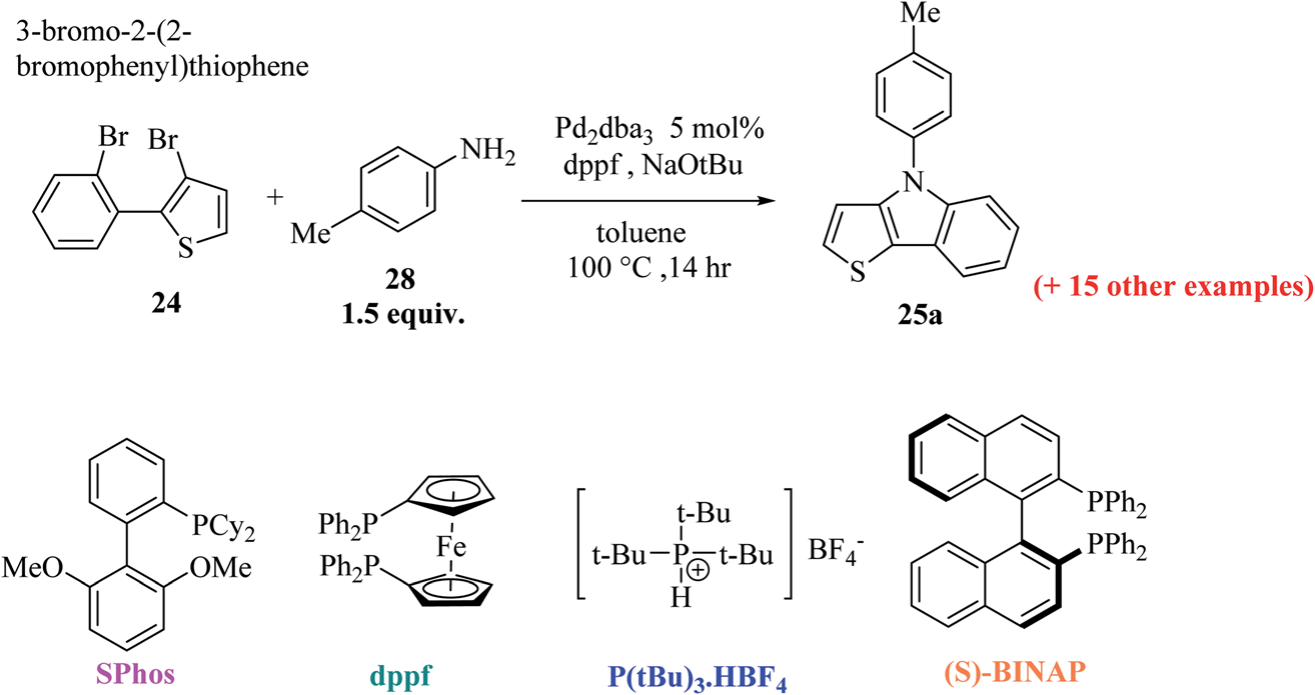
Pd-Catalyzed C–N coupling of 3-bromo-2-(2-bromophenyl)thiophene 24 to give 25a.

Further, 3-bromo-2-(2-bromophenyl)thiophene 24 undergoes Pd-catalyzed two-fold C–N coupling with *p*-methyl aniline 28 to give 25a (Scheme 8). Four ligands were screened using NaO*t*Bu in toluene and Pd_2_(dba)_3_ to enhance the yield of 25a and it was observed that the use of bidentate ligands dppf and (S)-BINAP gave 25a in 97% and 67% yield, respectively, whereas the bulky monodentate ligands SPhos and P(*t*Bu)_3_·HBF_4_ gave 25a in 65% and 86% yield, respectively. However, the bidentate ligand DPPF was found to give the highest yield,^21^*i.e.*, 97% (Table 1).

**Table 1:** Results of ligand screening

S. No.	Ligand [mol%]	Yield (%)
1	P(*t*Bu)_3_·HBF_4_[10%]	86
2	dppf [5%]	97
3	SPhos [10%]	65
4	(S)-BINAP[5%]	67

**Fig. 5:**
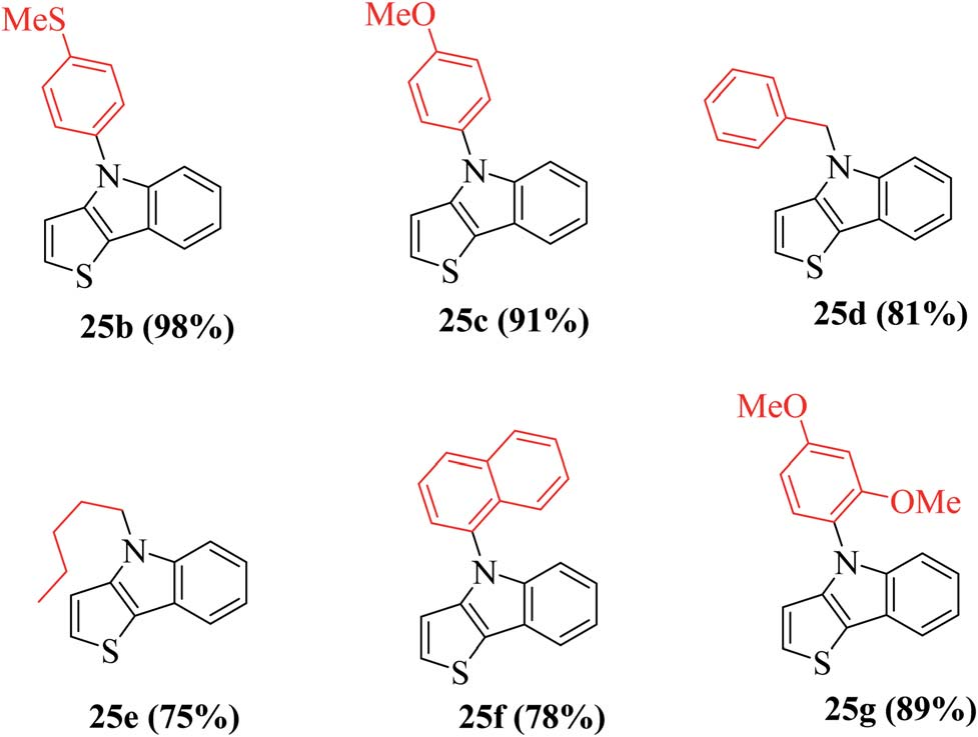
Yield of products having different substituents.

Using these optimized conditions, compound 24 was treated with electron-rich amines, namely, 4-(methylthio)aniline and *p*-anisidine, which produced the corresponding thienoindoles 25b and 25c in excellent yields. When electron-poor, benzylic and aliphatic amines were used, 1,1′-bis(diphenylphosphino)ferrocene (dppf) resulted in poor yield of the products. Thus, dppf was replaced by (S)-BINAP, which gave the corresponding thienoindoles 25d and 25e in good yields. Treatment of 1-naphthylamine gave the product 25f in a comparatively low yield (78%), whereas sterically hindered amine gave the product 25g in very good yield. Hence, the maximum yield was observed for electron-rich amines. In total, 20 substituted thieno[3,2-*b*]indoles were synthesized using this method, some of which are shown in Fig. 5.

**Scheme 9:**
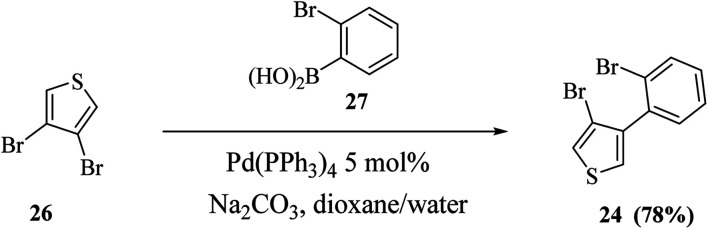
Site-selective Suzuki cross-coupling of 3,4-dibromothiophene.

Also, thieno[3,4-*b*]indoles were synthesized *via* the site-selective Suzuki cross-coupling reaction of 3,4-dibromothiophene 26 to form 3-bromo-4-(2-bromophenyl)thiophene 24 in 78% yield (Scheme 9).

**Scheme 10:**
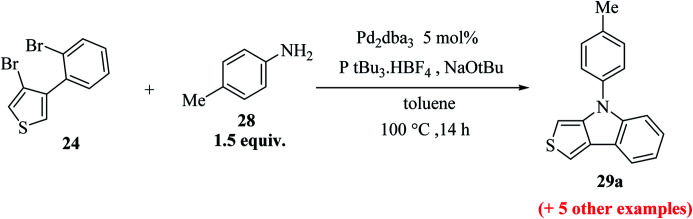
Two-fold C–N coupling of compound 24.

Further, 3-bromo-4-(2-bromophenyl)thiophene 24 undergoes Pd-catalyzed two-fold C–N coupling with *p*-methyl aniline 28 to give 29a (Scheme 10).

Upon screening of various ligands, the monodentate ligand P(*t*Bu)_3_.·HBF_4_ was found to be the most effective, giving the corresponding thieno[3,4-*b*]indole 29a in 95% yield (Table 2).

**Table 2:** Results of ligand screening

S. No.	Ligand [mol%]	Yield (%)
1	P(*t*Bu)_3_·HBF_4_[10%]	95
2	dppf [5%]	43
3	SPhos [10%]	65
4	(S)-BINAP[5%]	36

**Scheme 11:**
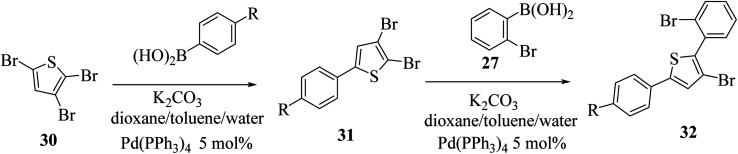
Coupling reaction of 2,3,5-tribromothiophene 30 to give compound 32.

At last, the practicality of the strategy was examined for the preparation of 5-substituted thieno[3,2-*b*]indoles. The synthesis began with two successive site-selective Suzuki cross-coupling reactions of 2,3,5-tribromothiophene 30 utilising the earlier reported conditions. 2,3,5-Tribromothiophene was initially converted to 2,3-dibromo-5-arylthiophene 31a–d, and later to 3-bromo-2-(2-bromophenyl)-5-arylthiophene 32a–d (Scheme 11), giving product 33 in low yield. This is presumably because of the extra aryl group at position-5, which may affect the overall electronic nature^22^ of the molecule (Table 3).

**Table 3:** Percentage yield of 31 and 32 using various R groups

R	31 (yield%)	32 (yield%)
(a) H	60	43
(b) Cl	37	30
(c) F	43	33
(d) *t*-Bu	43	26

**Scheme 12:**
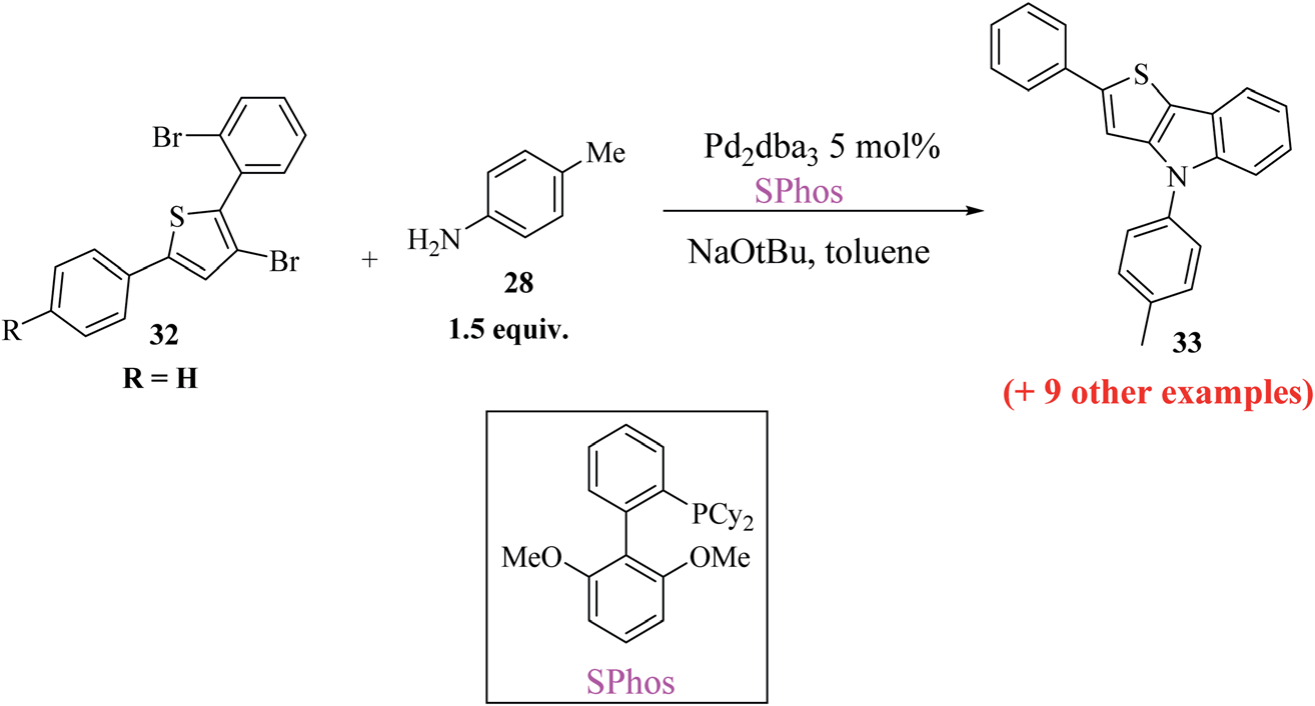
Synthesis of 5-substituted thieno[3,2-*b*]indole 33.

Buchwald's biaryl ligand SPhos gave the final product 33a in the highest yield, *i.e.*, 96% (Scheme 12), whereas ligands such as (*t*-Bu)_3_P·HBF_4_, dppf and xantphos gave compound 33a in 84%, 36% and 43% yield, respectively.

In conclusion, thieno[3,2-*b*]indoles and thieno[3,4-*b*]indoles have been synthesized *via* a new, more efficient and convenient synthetic methodology, namely, Buchwald–Hartwig cross-coupling. Also, the role of the ligand was found to be more crucial in the second step.

### 2.4 Synthesis of thieno[3,2-*b*]indole *via* Cadogan reductive cyclization

**Scheme 13:**
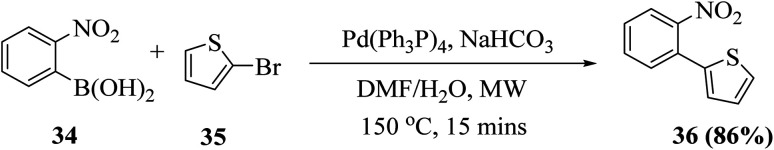
Synthesis of 2-(2-nitrophenyl)thiophene 36*via* Suzuki–Miyaura coupling reaction.

Dehaen *et al.*^19^ synthesized thieno[3,2-*b*]indole in two steps. The first step is the Suzuki–Miyaura coupling reaction between *o*-nitrophenyl boronic acid 34 and 2-bromothiophene 35. The recent literature revealed that arylboronic acids substituted with electron-withdrawing groups (here, nitro at the ortho position) undergo extensive deboronation under standard Suzuki–Miyaura conditions, which employ aq. Na_2_CO_3_ as the base. This premature destruction of the C–B bond causes low yields. Hence, to reduce or protect from proto-deboronation, Suzuki–Miyaura coupling reaction has been performed under microwave-enhanced conditions. The second step is the nitrene-mediated reductive cyclization of 2-(2-nitrophenyl)thiophene 36 under MW irradiation, which leads to a dramatic rate enhancement given that the usual method demands drastic conditions and long reaction time (Scheme 13).

**Scheme 14:**
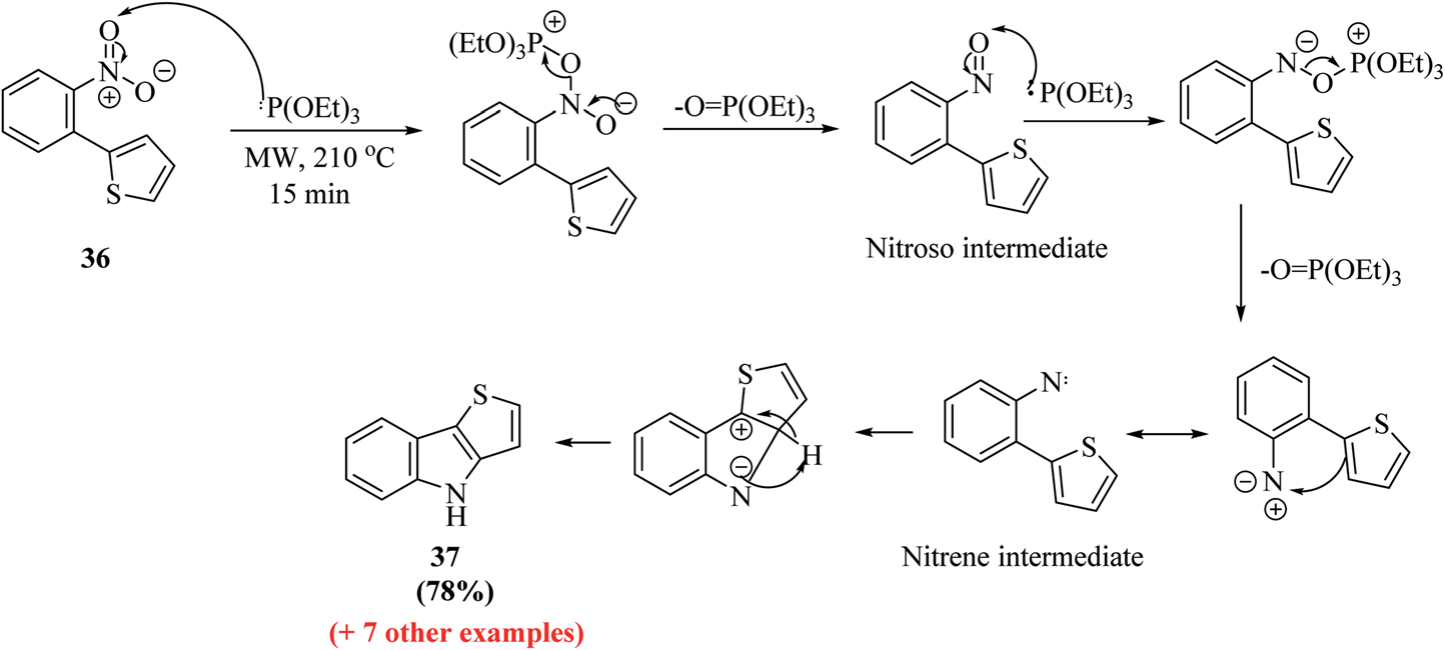
Cadogan reductive cyclization to thieno[3,2-*b*]indole 37.

Further, a mixture of compound 36 and triethyl phosphite was suspended in a 10 mL sealed glass vial and irradiated with 300 W power at 210 °C. Bunyan and Cadogan demonstrated that aromatic *C*-nitroso-compounds are promptly deoxygenated by triethyl phosphite. Hence, deoxygenation of 36 led to the formation of a nitroso-compound, which readily underwent deoxygenation, resulting in the formation of an indole ring *via* a nitrene intermediate.^23^ The reaction took 15 min to go to completion and acidic work-up removed the phosphate by-products and product 37 was obtained in good yield (Scheme 14).

Dehaen *et al.* used 2-nitro-phenylboronic acid given that it gives access to the biaryl compounds needed for the Cadogan cyclization and also heterocyclic boronic acids are expensive and difficult to synthesize. Hence, it is a more versatile, efficient and economic method for synthesizing thieno[3,2-*b*]indoles.

### 2.5 Metal-free approach for synthesizing regioselective thieno[2,3-*b*]indole

**Scheme 15:**
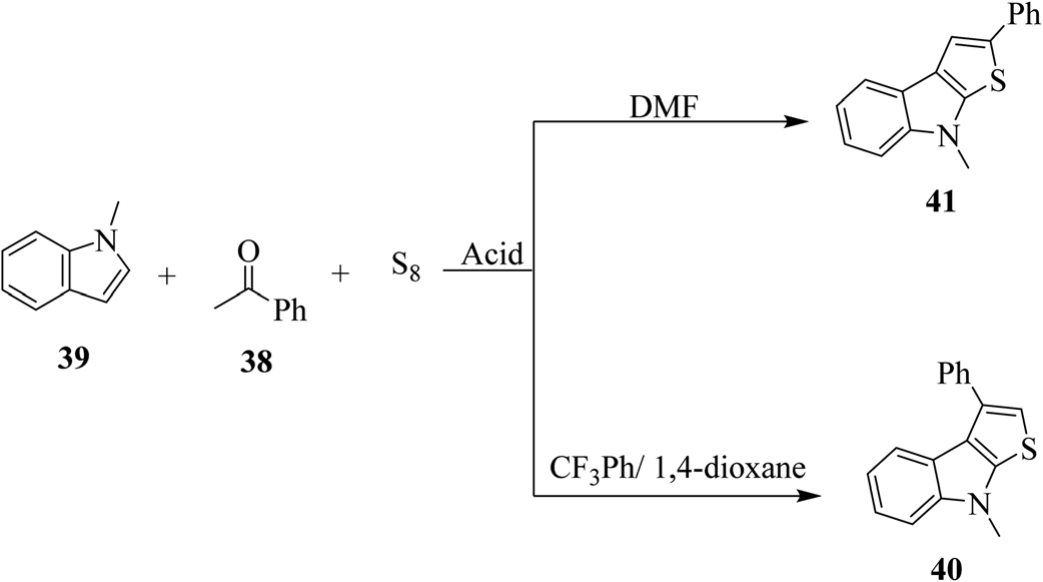
Methodology for synthesizing benzothieno[2,3-*b*]indole.

Penghui *et al.*^24^ described an effective metal-free approach for synthesizing substituted thieno[2,3-*b*]indole 40 and 41 with high regioselectivity and great functional group tolerance. In this approach, the cascade cyclization occurs *via* the acid-promoted annulation of ketone 38, indole 39 and sulfur powder, where the solvent DMF and the additive control the regioselectivity of the reaction (Scheme 15).

#### 2.5.1 Multi-component reactions

Multicomponent reactions are very efficient given that they are an easy and atom-economic approach, which is highly advantageous compared to the conventional methods of synthesis. In this process, more than two starting materials combine to form a product, which contains almost all the employed atoms. MCRs can be divided into three types, *i.e.*, domino or cascade, sequential and consecutive MCR. Domino reactions take place without the requirement of additional reagents or without the need for changing the reaction conditions, *i.e.*, everything needed for the reaction is there at the beginning. In the case of sequential MCR, the functionality necessary for the second step is created in the first step but an additional reagent must be added for the second reaction to occur. In consecutive MCR, the subsequent addition of reagent is done together with changing the reaction conditions from one step to another. Each type of MCR provides high structural and functional diversity.^25,26^

**Scheme 16:**
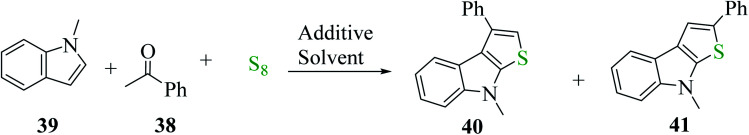
Schematic representation of the synthesis of 3-phenylthieno[2,3-*b*]indole 40 and 2-phenylthieno[2,3-*b*]indole 41.

Elemental sulfur was used as the source of sulfur given that it is abundant in nature, non-toxic and stable under normal conditions. Its low price and high purity make it a good choice. In recent years, elemental sulfur had found great applications in C–S bond-forming reactions. Most of the reported C–S bond formation reactions using elemental sulfur are catalyzed by transition metals. However, a few reactions are also available that do not need any transition metal.^27^ Our reaction is among these types of reactions (Scheme 16).

1-Methyl-1*H*-indole 39, acetophenone 38 and sulfur powder were reacted using different additives and solvents. The desired product, *i.e.*, 3-phenylthieno[2,3-*b*]indole 40 was obtained when chlorobenzene was used as the solvent and 50 mol% HI as an additive at 130 °C. Further, the yield of product 40 was improved using PhCF_3_ and anisole as additives and 1,4-dioxane as the solvent. The yield of 40 was further improved using PhCF_3_/1,4-dioxane, which was further greatly enhanced when 1 equivalent of l-phenylalanine was used together with PhCF_3_ and 1,4-dioxane. 2-Phenylthieno[2,3-*b*]indole 41 was obtained when DMF was chosen as the solvent, whose yield was increased when acetic acid was used as the acid instead of HI. This is because when DMF was used as the solvent, the regioselectivity of the cyclization process switches as a result of the change in the polarity of solvent. This is a direct cyclization reaction. The reaction yield of 41 and the ratio of 41 : 40 further increased when the temperature was increased to 150 °C and the ratio of 39 : 38 changed to 1 : 2.3.

**Scheme 17:**
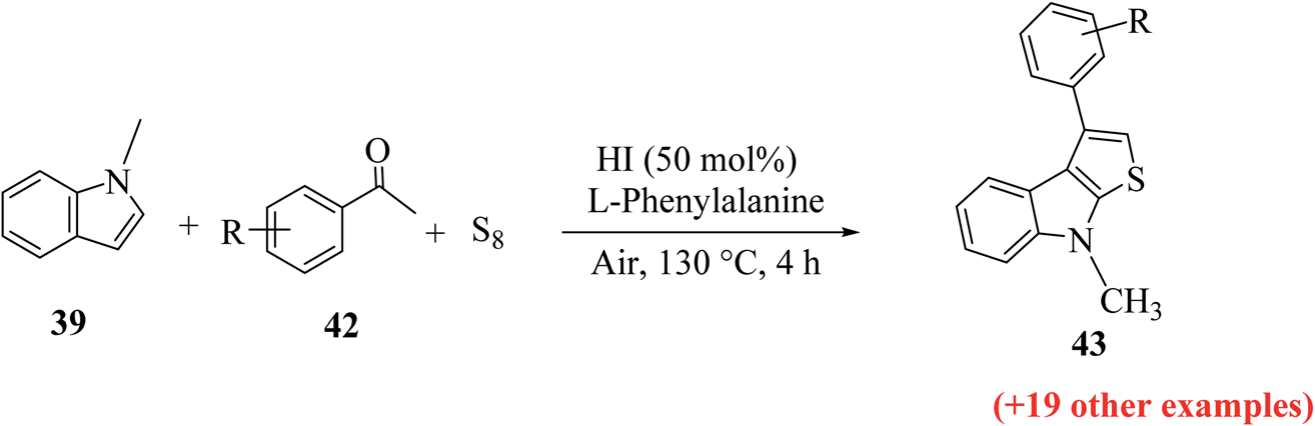
Synthesis of 3-substituted thieno[2,3-*b*]indole analogs 43 using different substituted indoles 42.

#### 2.5.2 Substrate scope of 3-substituted thieno[2,3-*b*]indole synthesis

**Fig. 6:**
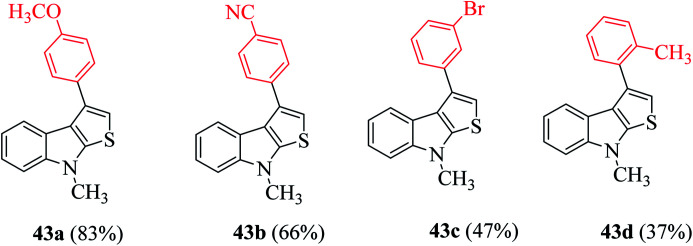
Analogs of 3-substituted thieno[2,3-*b*]indole.

Product 43 was obtained in moderate to excellent yield (31–83%) using various substituted indoles (Scheme 17). 43a was formed in 83% when the substrate had a methoxy group as the substituent and 66% of 43b was formed when the substituent was the cyano group. Bulky substrates such as 1-(3-bromophenyl)ethanone and 1-(*o*-tolyl)ethanone gave 47% of 43c and 37% of 43d, respectively, due to steric hindrance (Fig. 6).

**Scheme 18:**
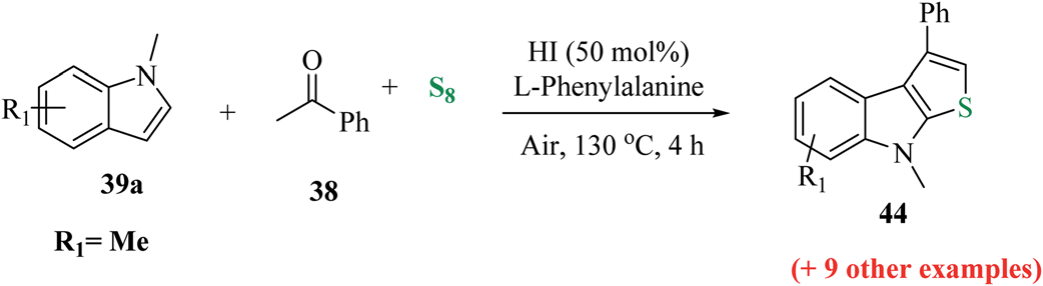
Synthesis of 3-substituted thieno[2,3-*b*]indole analogs by varying the position of the functional group on the indole ring.

Further, the yield of product 44 was affected by the position of the functional group on the indole ring. When it was present on C-5, C-6 and C-7, then the yield of the product was good, whereas when it was present at the C-4 position, the yield decreased dramatically (Scheme 18).

#### 2.5.3 Substrate scope of 2-substituted thieno[2,3-*b*]indole synthesis

**Scheme 19:**
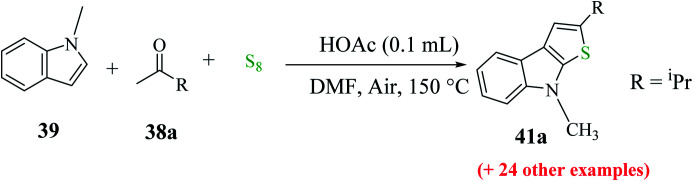
Synthesis of 2-substituted thieno[2,3-*b*]indole analogs using different substituted ketones.

41a was synthesized using different ketones in yield of up to 85%. The product was obtained in good yield when aromatic acetophenones were used, irrespective of the position of the functional group, whereas aliphatic ketones such as ^i^PrCOCH_3_38a gave 2-isopropyl-8-methyl-8*H*-thieno[2,3-*b*]indole 41a in moderate yield (Scheme 19).

**Scheme 20:**
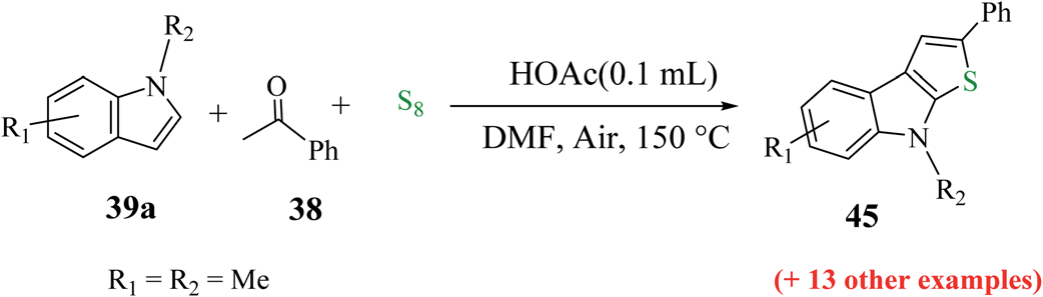
Synthesis of 2-substituted thieno[2,3-*b*]indole analogs using different indoles.

Further, indoles bearing various substituents gave the product in a yield of up to 83%. When Me was at the C-6 or C-7 position of the indole moiety, the yield of product 45 decreased slightly to 67% and 73%, respectively, as shown in Scheme 20.

#### 2.5.4 Scheme for preparing 3-phenylthieno[2,3-*b*]indole

**Scheme 21:**
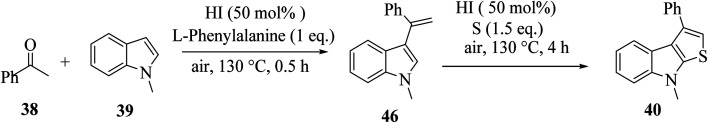
Schematic representation of 3-phenylthieno[2,3-*b*]indole synthesis.

When 1-methyl-indole reacted with acetophenone in the absence of elemental sulfur, it gave 1-methyl-3-(1-phenylvinyl)-1*H*-indole 46 as an intermediate, which on reaction for 4 h with HI and sulfur powder in air at 130 °C, gave 3-phenylthieno[2,3-*b*]indole 40 in 95% yield. This reaction proceeded *via* a [4 + 1]-type synthetic route to give our product^28^40 (Scheme 21).

#### 2.5.5 Mechanism for 2-phenylthieno[2,3-*b*]indole synthesis

**Scheme 22:**
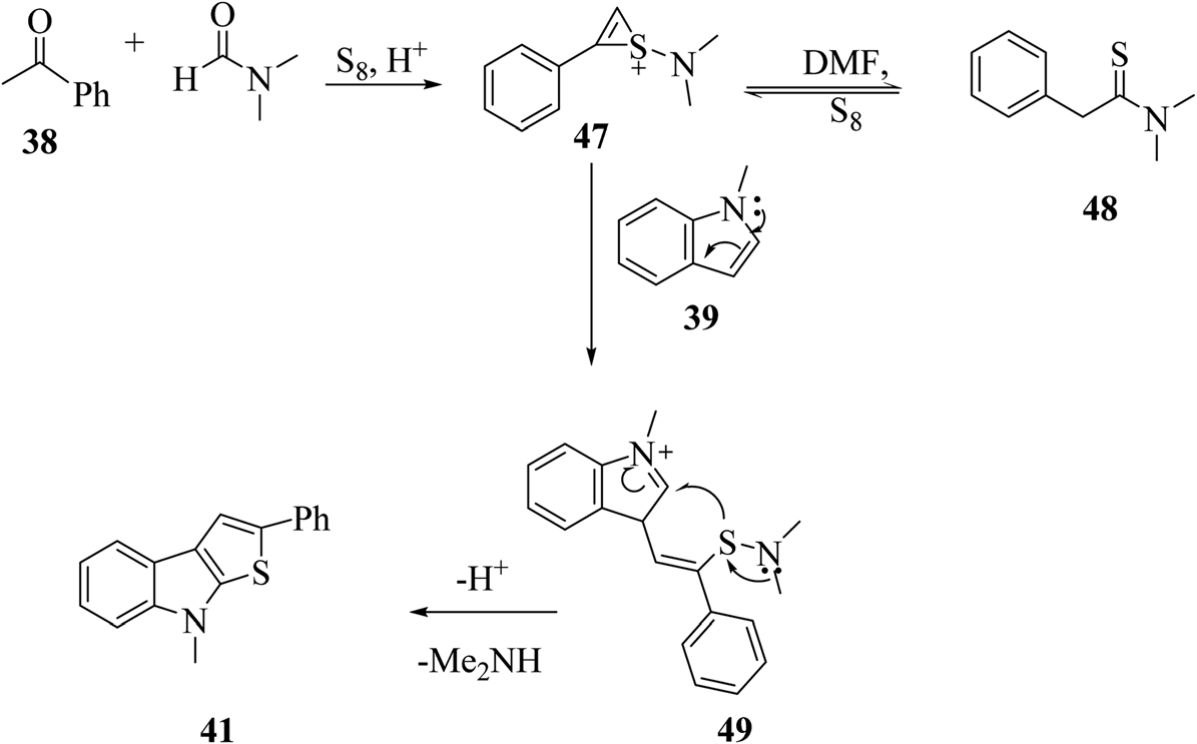
Mechanism for the formation of 2-phenylthieno[2,3-*b*]indole.

When acetophenone 38 was treated with sulfur powder in dry DMF under acidic conditions, *N*,*N*-dimethyl-2-phenylethanethioamide 47 was obtained (Willgerodt–Kindler reaction),^29,30^ which resonates to form the intermediate *N*,*N*-dimethyl-2-phenylethanethioamide 48. Moreover, 47 reacted with 1-methyl-1*H*-indole to give intermediate 49, which was converted to 2-phenylthieno[2,3-*b*]indole 41 under acidic conditions (Scheme 22).

### 2.6 Three-component metal free synthesis of thieno[2,3-*b*]indole in sulfur powder

**Scheme 23:**
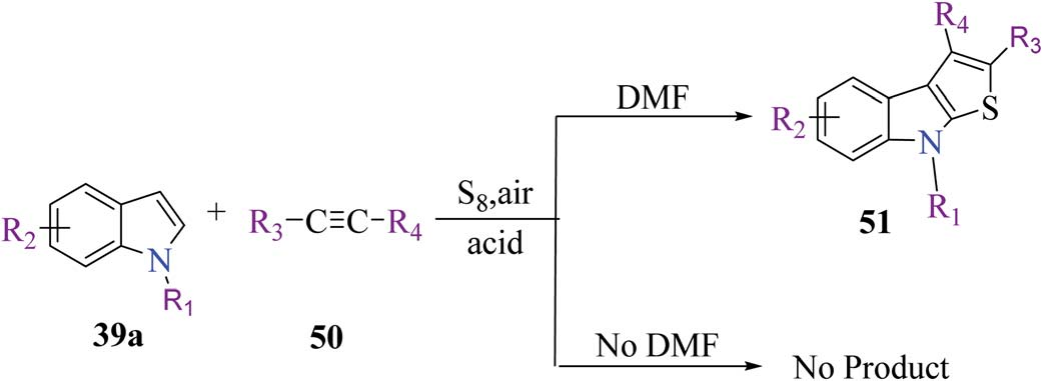
Strategy for the synthesis of indole-fused heterocycle.

Li *et al.*^31^ synthesized thieno[2,3-*b*]indole from indoles and alkenes or alkynes in the presence of sulfur powder and in the absence of metal. This is a simple and efficient method in which a Brønsted acid promotes the formation of substituted thieno[2,3-*b*]indole, where DMF is essential for converting the reactants into the fused products. Substituted 1-methylindole 39a, substituted alkyne 50 and sulfur powder were treated in acid at 150 °C and in metal-free conditions using DMF as a solvent to obtain product 51. However, in the absence of DMF, no product was obtained (Scheme 23).

**Scheme 24:**
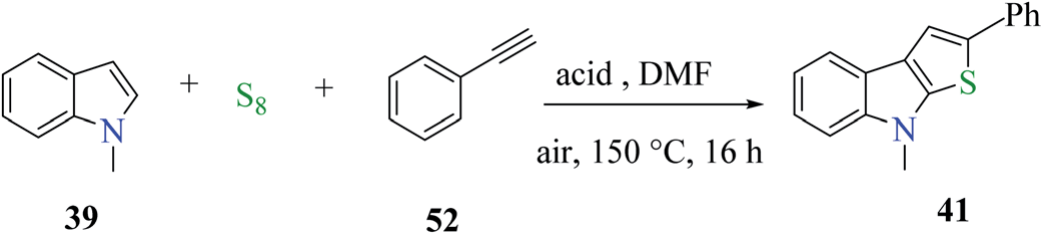
Optimization of the reaction conditions.

Further, product 41 was obtained in 70% yield when AcOH was used as an acid. Moreover, inorganic acids such as hydrochloric acid acted as the most efficient acid and gave the product in 86% yield. Reducing either the reaction temperature or HCl concentration reduced the yield of the product (Scheme 24).

#### 2.6.1 Substrate scope for the formation of substituted thieno[2,3-*b*]indole

**Scheme 25:**
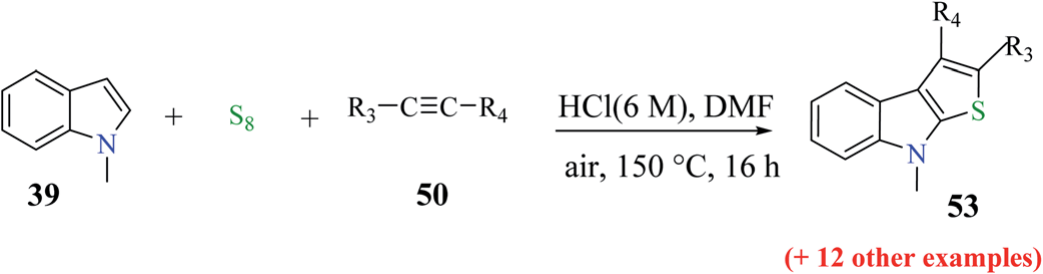
Synthesis of thieno[2,3-*b*]indole analogs by varying the substituents on the alkyne.

The yield of the product depends on the substrate used (Scheme 25).

**Fig. 7:**
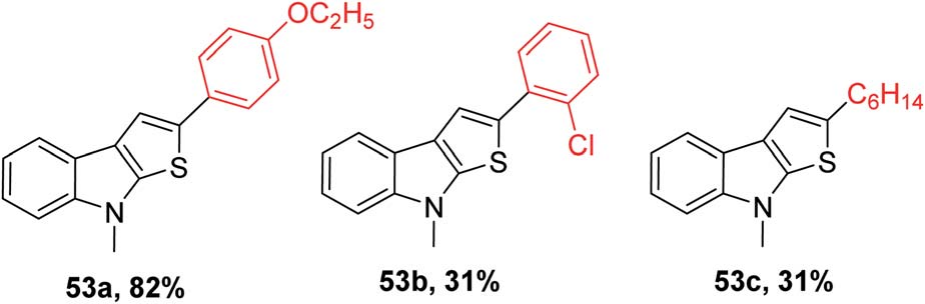
Analogs of 2-substituted thieno[2,3-*b*]indole.

53 was obtained in 31% to 82% when substituted alkynes were used. *Para*-substituted phenylacetylene such as *p*-ethoxyphenylacetylene reacted with 1-methylindole 39 in the presence of sulfur powder to give the product 2-(4-ethoxyphenyl)-8-methyl-8*H*-thieno[2,3-*b*]indole 53a in 82% yield. When 1-chloro-2-ethylbenzene was used as the substrate, 31% of 2-(2-chlorophenyl)-8-methyl-8*H*-thieno[2,3-*b*]indole 53b was formed due to the steric hindrance caused by the substrate and when aliphatic alkynes such as 1-octyne were used as the substrate, 31% of 2-hexyl-8-methyl-8*H*-thieno[2,3-*b*]indole 53c was formed because aliphatic alkynes do not favour annulation (Fig. 7).

**Scheme 26:**
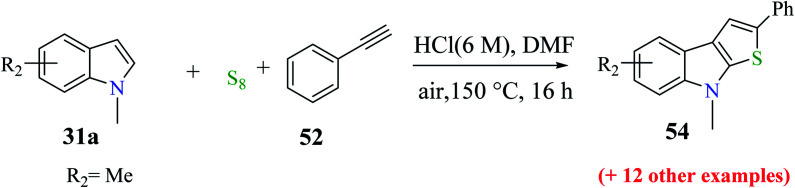
Extension of the substrate scope with respect to indoles.

Further, different substituted indoles 39a were reacted with phenylacetylene 52 and sulfur powder and the product was obtained in yields of up to 81%. When methyl was substituted at the C-5 position of 1-methyl-1*H*-indole, 61% of 5,8-dimethyl-2-phenyl-8*H*-thieno[2,3-*b*]indole was obtained, and when it is substituted at the C-7 position, 56% of 7,8-dimethyl-2-phenyl-8*H*-thieno[2,3-*b*]indole was formed (Scheme 26).

**Scheme 27:**
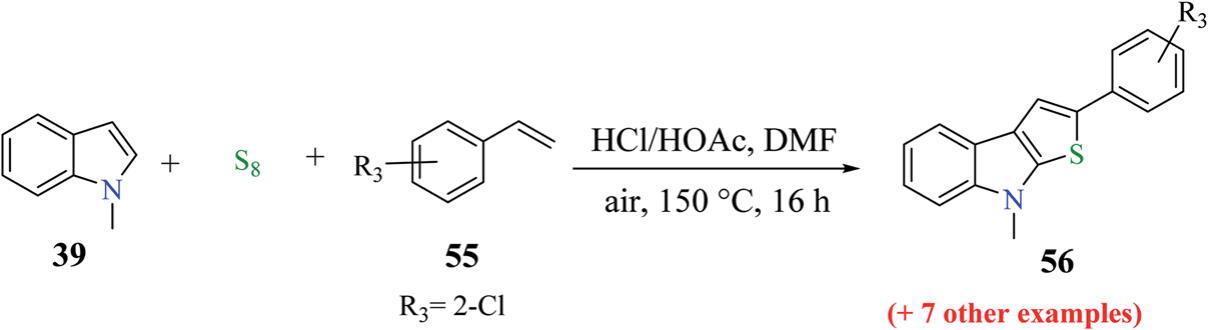
Synthesis of thieno[2,3-*b*]indoles using different substituted styrenes.

Moreover, 1-methylindole 39 was reacted with substituted styrene 55 and sulfur powder as the source of sulfur to get up to 76% of 2-substituted thieno[2,3-*b*]indole. Styrene derivatives with either an electron-donating group such as methoxy and methyl, or halogen (F, Cl or Br) gave the product in good yield upon reacting with 1-methylindole. Overall, the reaction yield was influenced by the placement of the substituent on the phenyl motif of styrene. The use of 1-chloro-2-vinyl benzene as the substrate gave product 56 in 30% yield (Scheme 27).

**Scheme 28:**
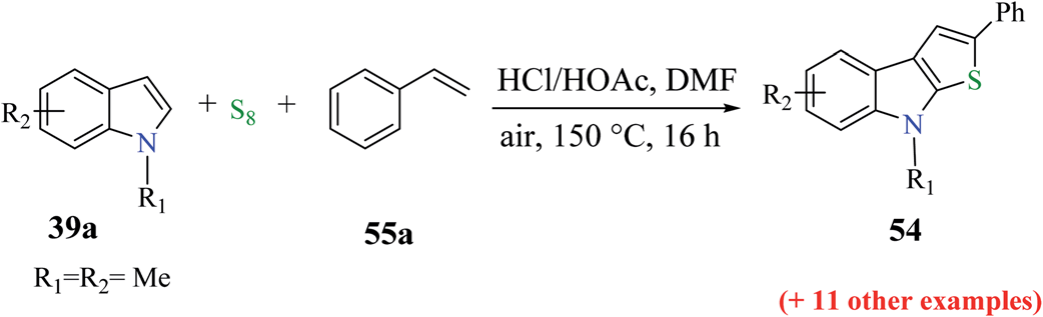
Reaction of different substituted indoles with styrene under the optimized reaction conditions.

The reactions of substituted indoles 39a such as methyl, methoxy, bromo, fluoro and chloro gave the product in good yield independent of the position of the substituent at the C-5, C-6 or C-7 position. Even good yield of the product was obtained when unprotected indole was used as the substrate (54–79%, Scheme 28).

**Scheme 29:**
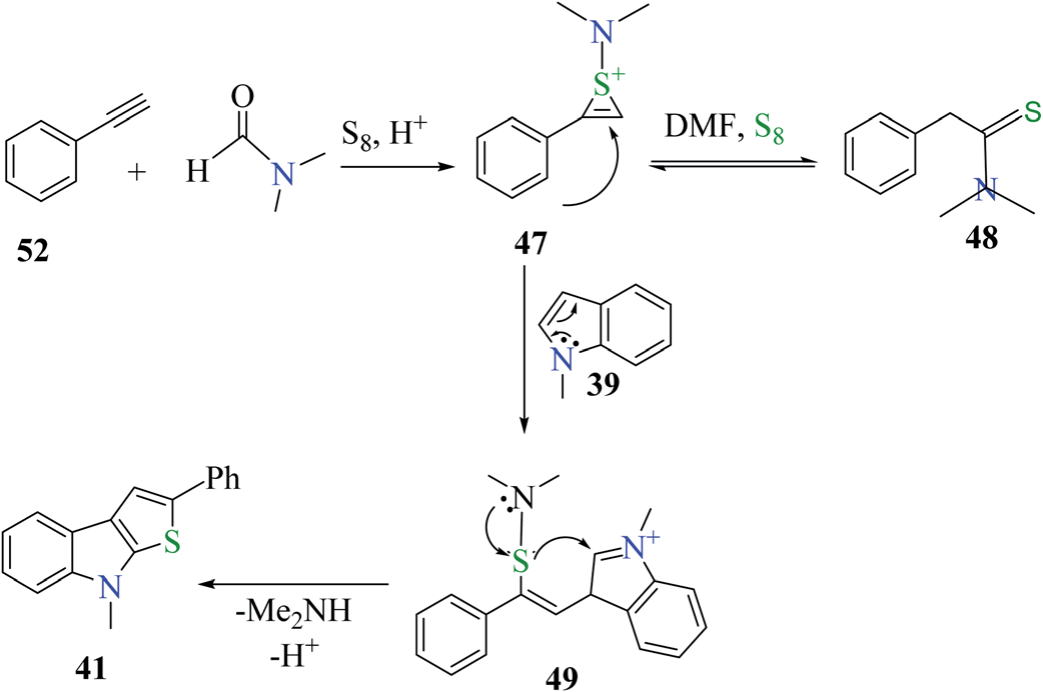
Proposed mechanism for the formation of thieno[2,3-*b*]indole derivative 41.

Plausible mechanism involves the reaction of phenylacetylene 52 with DMF and sulfur powder to form intermediate 47, which resonates to form 48. Further, 47 reacts with 1-methyl-indole 39 to form 49, which ultimately gets converted to 8-methyl-2-phenyl-8*H*-thieno[2,3-*b*]indole 41, the desired product^32,33^ in moderate yield (Scheme 29).

### 2.7 Preparation of thieno[3,2-*b*]indole by halogen dance and sequential coupling reaction

Hayashi *et al.*^34^ reported the synthesis of thieno[3,2-*b*]indole from 2,5 dibromothiophene 57 and lithium diisopropylamide (LDA), which resulted in the formation of transient thienyl anion species 58*via* the mediated halogen dance reaction,^35^ which led to the development of two chemical bonds in one pot, followed by Negishi coupling^36^ and Suzuki–Miyaura coupling or Buchwald–Hartwig amination *via* tandem catalysis in the presence of 1,1′-bis(diphenylphosphino)ferrocene (dppf) and tri-*tert*-butylphosphine (*t*Bu_3_P) ligand to give thieno[3,2-*b*]indole.

**Scheme 30:**
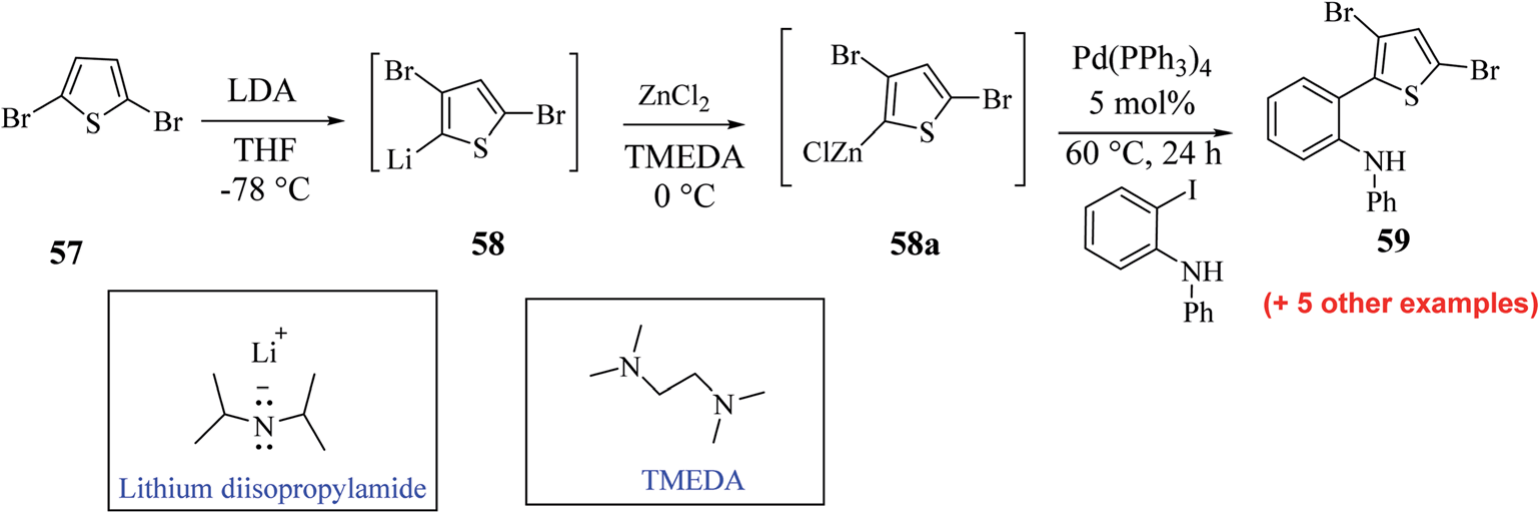
Synthetic route for 2-(3,5-dibromothiophen-2-yl)-*N*-phenylaniline.

The favourable environment for halogen dance with 2,5-dibromothiophene 57 occurred by its deprotonation at the 4-position with 1.3 eq. LDA at −78 °C for 5 min.^37^ Moreover, the lithiated species gets rearranged to 5-lithio-4-brominated thiophene 58 at −78 °C *via* halogen dance, which on treatment with ZnCl_2_ in tetramethylethylenediamine^38^ (TMEDA 1.4 equiv.) at 0 °C formed thienylzinc species 58a, which was further subjected to coupling conditions using the transition metal catalyst Pd(PPh_3_)_4_ and protected iodoaniline at 60 °C for 24 h, forming the coupled product 59 in 69% yield (Scheme 30).

**Scheme 31:**
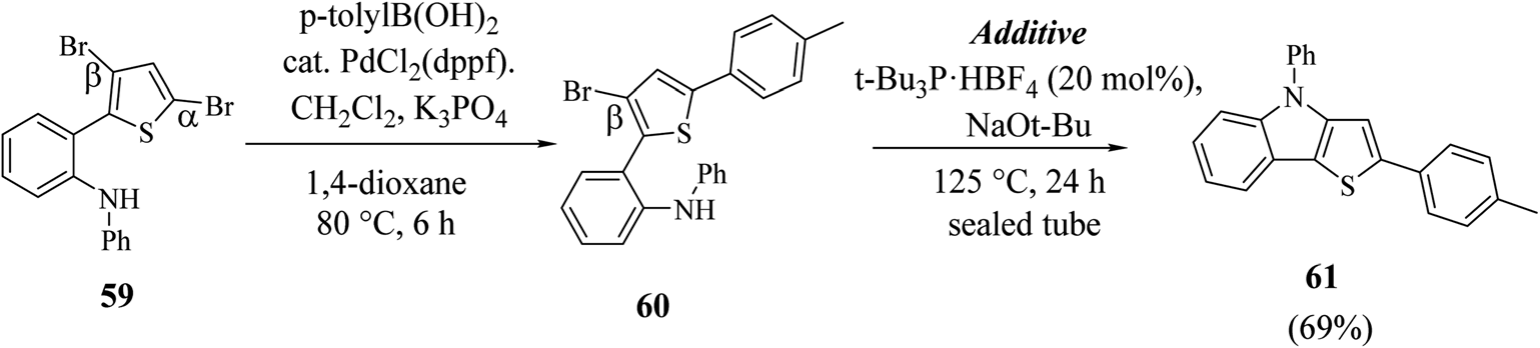
Synthetic route for the stepwise Suzuki–Miyaura coupling and intramolecular Buchwald–Hartwig amination reaction.

Hayashi *et al.*^34^ reported the synthetic approach of 59 towards ligand-controlled Suzuki–Miyaura coupling and Buchwald–Hartwig amination *via* assisted tandem catalytic transformation. Here, 2-(3,5-dibromothiophen-2-yl)-*N*-phenylaniline 59 gave π-conjugated thienoindole *via* Suzuki–Miyaura coupling^39,40^ and C–N bond formed by Pd-catalyzed reaction in the presence of aryl halide and amine groups in a stoichiometric amount of base (intramolecular Buchwald–Hartwig amination).^41–43^ Arylation at the α-position of thiophene resulted in the formation of 2-(3-bromo-5-(*p*-tolyl)thiophen-2-yl)-*N*-phenylaniline 60 and amination of the remaining β-bromo group with 2-aminophenyl group led to the formation of 4-phenyl-2-(*p*-tolyl)-4*H*-thieno[3,2-*b*]indole 61 (Scheme 31).

**Scheme 32:**
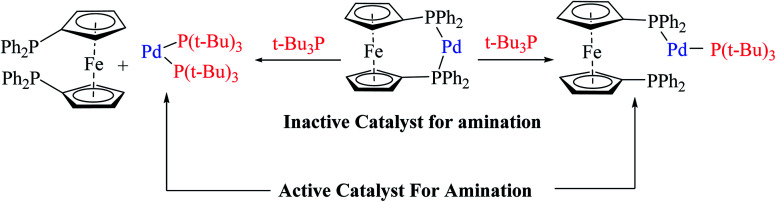
Action of additive *t*-Bu_3_P on activity of the Pd-dppf catalyst for amination.

The above-mentioned reaction failed in the absence of additive, as reported by Okano and coworkers,^44^ demonstrating that the additive plays an important role in significant amination for getting the desired product. Despite the longer response time of 24 h at 125 °C, additives such as NaO*t*Bu were detected to be insufficient. The addition of *t*Bu_3_P·HBF_4_ (20 mol%) notably promoted amination to form 4-phenyl-2-(*p*-tolyl)-4*H*-thieno[3,2-*b*]indole 61 in 69% yield. A reduced amount of the phosphorus ligand in the additive led to a lower yield of 61. This outcome suggests that *t*Bu_3_P, a monodentate ligand, needs to coordinate Pd with dppf, a bidentate ligand to form the efficient catalyst *in situ* for intramolecular amination (Scheme 32).

**Scheme 33:**
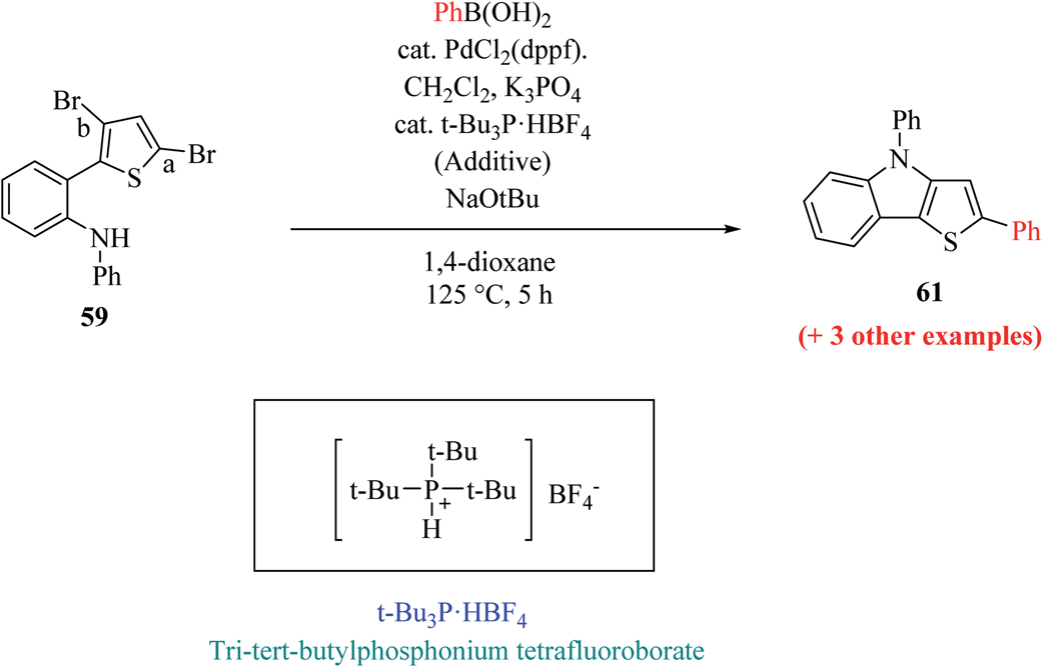
Synthetic route for one-pot single-shot addition.

The one-pot reaction was also reported at 125 °C for 5 h by one-shot addition, which included all the required reagents for forming aryl substituted thieno[3,2-*b*]indole (Scheme 33).

A few of the limitations of the above-mentioned one-pot reaction were justified based on the variation in the Ar group at the second position of thieno[3,2-*b*]indole 61. The above-mentioned reaction conditions (Scheme 20) were not applicable for Ar = 4-nitrophenylboronic acid given that it has low solubility in 1,4-dioxane, and thus water as the co-solvent in a ratio of 4 : 1 mainly helps to maximize the yield of 2-(2-nitrophenyl)-4-phenyl-4*H*-thieno[3,2-*b*]indole (Ar = 2-nitrophenyl) to 43%.

**Scheme 34:**
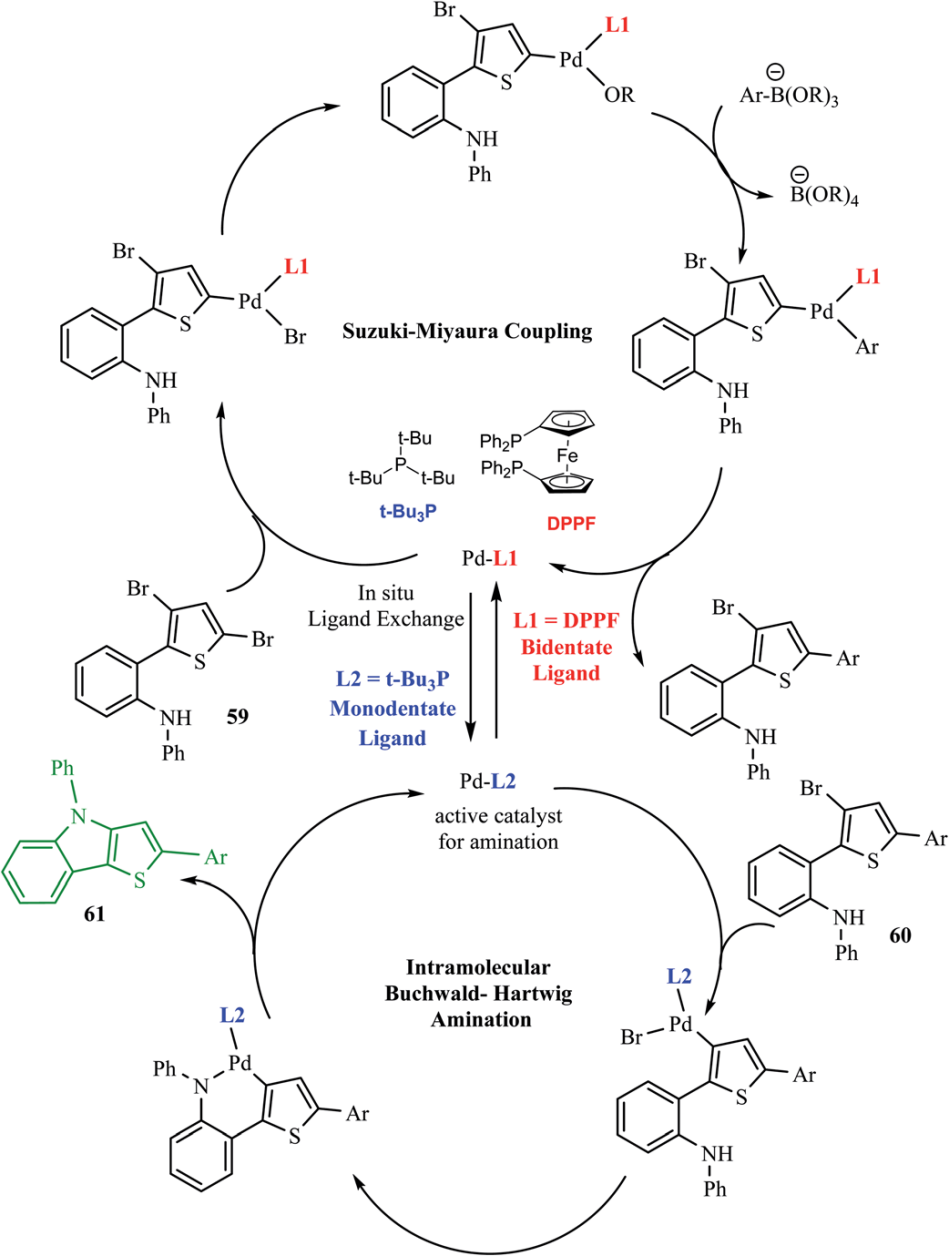
Tandem catalytic pathway for sequential coupling reaction.

The Pd-dppf catalyst plays an important role in Suzuki–Miyaura coupling, which led to C–C bond formation at the α-position of thiophene by releasing the bromo group. Further, ligands were exchanged from Pd-dppf to Pd-(*t*Bu_3_P)_2_ and the reaction moves towards C–N bond formation in which the β-bromo group undergoes oxidative addition of the Pd-(*t*Bu_3_P)_2_ catalyst and reductive elimination to yield 4-phenyl-2-(aryl)-4*H*-thieno[3,2-*b*]indole 61. The maximum yield was obtained when *t*Bu_3_P was used as an additive for amination. The following sequential coupling reactions (Scheme 34) were reported as tandem catalytic pathways.^34^

### 2.8 Synthesis of 2-(hetero)aryl-substituted thieno[3,2-*b*]indole *via* Fisher indolization

Irgashev *et al.*^45^ reported the Fischer indolization^46,47^ methodology for the development of 2-(hetero)aryl-substituted thieno[3,2-*b*]indole 69 from 5-(hetero)arylthiophen-3(2*H*)-ones 68 and phenyl hydrazine 62. The intermediate 5-(hetero)arylthiophen-3(2*H*)-one was acquired in two steps, *i.e.*, the reaction of α-bromocinnamates or their other hetero derivatives with methyl thioglycolate, followed by the base treatment of 5-(hetero)aryl-3-hydroxysubstituted 2-thenoates to yield 2-(hetero)aryl-substituted thieno[3,2-*b*]indole.

**Scheme 35:**
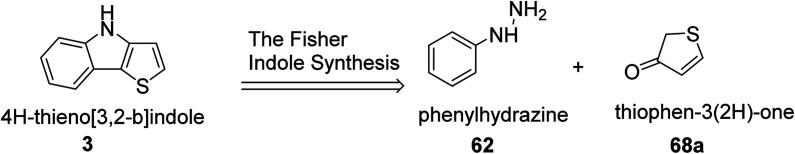
Retrosynthetic route of thieno[3,2-*b*]indole *via* Fisher indole synthesis.

Convenient and cheap synthetic approaches are urgent to meet the growing demand of hetero-arylated thieno[3,2-*b*]indoles, which are widely used in optoelectronic material engineering. In this context, the retro-synthetic approach towards the synthesis of thienoindoles is shown in the following scheme (Scheme 35).

**Scheme 36:**
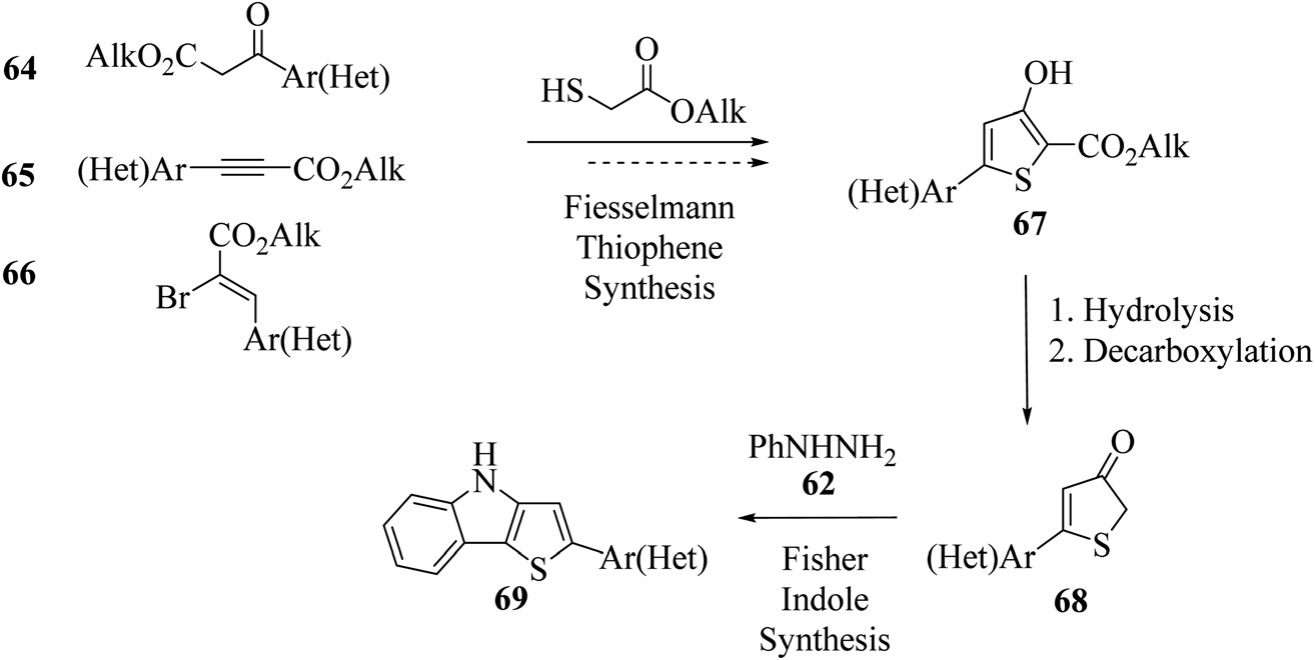
Synthetic route of 2-(hetero)aryl-substituted thieno[3,2-*b*]indoles *via* Fiesselmann and Fischer method.

An appropriate route for the synthesis of alkyl 3-hydroxythiophene-2-carboxylates (2-thenoates) *via* Fiesselmann thiophene synthesis^48^ was reported. It is a condensation reaction of 1,3-*C*,*C*-dielectrophilic substrates and alkyl thioglycolates upon treatment with base. 5-(Hetero)aryl-3-hydroxy-substituted 2-thenoates were synthesized in two steps starting from substrate 2-(hetero)aroylacetates^49^64 or 3-(hetero)arylpropiolates^50^65 or 2-bromo-3-(hetero)arylacrylates 66. Moreover, 2-bromo-3-(hetero)arylacrylates 66 were easily available and also act as a 1,3-dielectrophilic three-carbon center for the Fiesselmann method. Further, 68 was formed by ester hydrolysis and *in situ* decarboxylation of alkyl 3-hydroxythiophene-2-carboxylates 67 (Scheme 36).

**Scheme 37:**
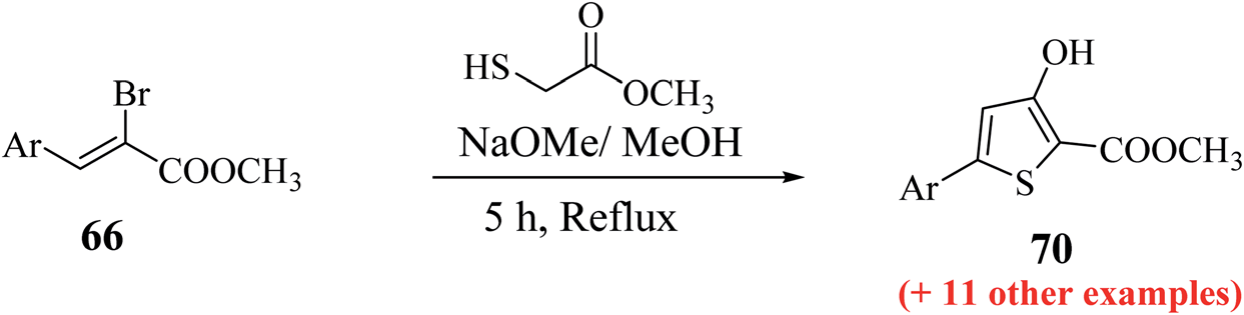
Synthetic route for methyl 5-aryl-3-hydroxythiophene-2-carboxylates.

The maximum yield of the product was reported when 2-bromo-3-(hetero)arylacrylate 66 and methylthioglycolate were slowly added to NaOMe base (4 equiv.) in dry MeOH and refluxing the reaction mixture for 5 h resulted in the formation of methyl-5-aryl-3-hydroxythiophene-2-carboxylates 70 (Scheme 37).

**Scheme 38:**
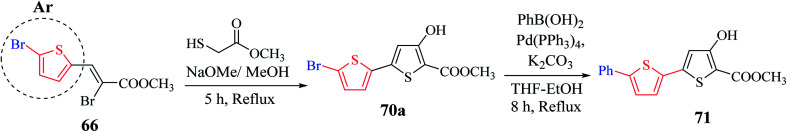
Synthetic route to methyl 4-hydroxy-5′-phenyl-[2,2′-bithiophene]-5-carboxylate 71.

Methyl 5′-bromo-4-hydroxy-[2,2′-bithiophene]-5-carboxylate 70a was synthesized from methyl-2-bromo-3-(5-bromothiophen-2-yl)acrylate 66a, which was in turn prepared *via* the esterification of 2-bromo-3-(5-bromothiophen-2-yl)acrylic acid^51^ (Scheme 38). The ester having a Br group was changed to 5-phenylthien-2-yl-connected derivative 71*via* Suzuki–Miyaura reaction.

**Scheme 39:**
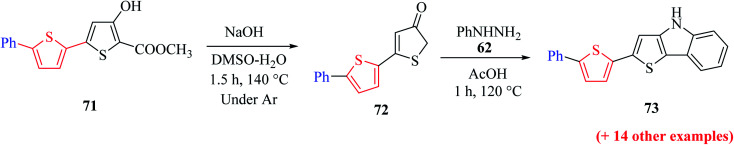
Synthetic approach towards the formation of 2-(5-phenylthiophen-2-yl)-4*H*-thieno[3,2-*b*]indole 73.

Further, saponification^45^ of methyl-4-hydroxy-5′-phenyl-[2,2′-bithiophene]-5-carboxylate 71 in the presence of NaOH and DMSO–H_2_O for 1.5 h at 140 °C under an argon atmosphere yielded 5′-phenyl-[2,2′-bithiophen]-4(5*H*)-one 72 in 94–95% yield, which on further treatment with phenylhydrazine 62 in glacial CH_3_COOH for 1 h at 120 °C, yielded 2-(5-phenylthiophen-2-yl)-4*H*-thieno[3,2-*b*]indole 73 in 58% yield (Scheme 39).

### 2.9 One-pot approach for synthesizing thieno[2,3-*b*]indole starting from indoline-2-thiones and nitroalkene-based adducts

**Scheme 40:**
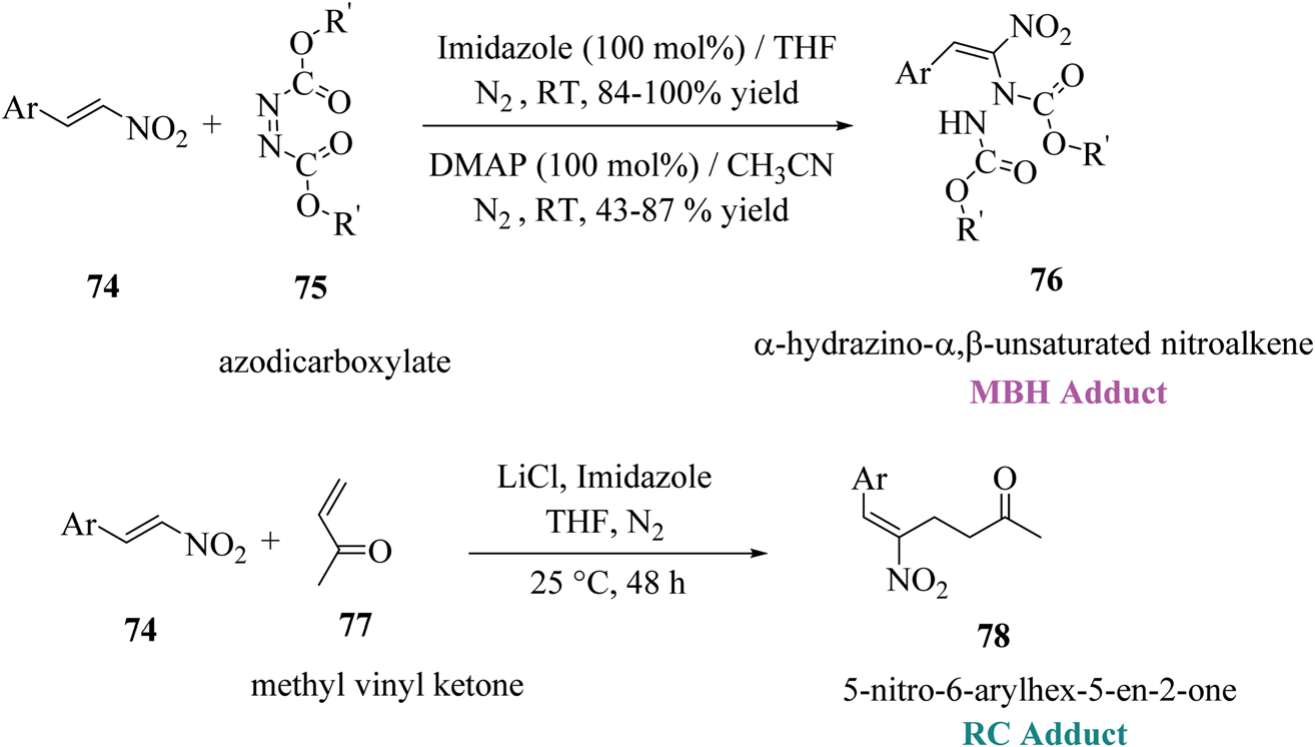
Synthesis of nitroalkene-based MBH and RC adducts.

Mane *et al.*^52^ reported a novel approach for the synthesis of thieno[2,3-*b*]indoles *via* the base-assisted [3 + 2]annulation of indoline-2-thione involving nitroalkene adducts, *i.e.*, Morita–Baylis–Hillman (MBH)^53,54^76 and Rauhut–Currier (RC)^55^78 adduct. The α-hydrazino-α,β-unsaturated nitroalkene intermediate was synthesized in excellent yields from imidazole or DMAP directed Morita–Baylis–Hillman reaction of nitroalkene and azodicarboxylates.^56^ Nitroalkene-based Rauhut–Currier adducts^57^ are effective synthons for synthesizing various functionalized heterocycles. Also, these synthons are great Michael acceptors, and also employed in Cascade Michael addition–cyclization reactions. The MBH adduct and RC adduct were synthesized, as shown in Scheme 40.

The reaction of 1-methylindoline-2-thione 79a and α-hydrazinonitroalkenes 76 was performed by using potassium acetate/acetic acid as an additive and CH_3_CN as the solvent to obtain diisopropyl-1-(8-methyl-3-(*p*-tolyl)-8*H*-thieno[2,3-*b*]indol-2-yl)hydrazine-1,2-dicarboxylate 80 in 78% yield.

**Scheme 41:**
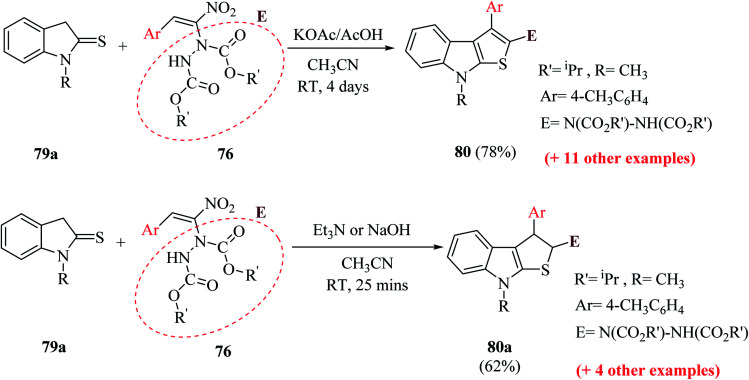
Synthesis of derivatives of thienoindole 80 and dihydrothienoindole 80a*via* MBH reaction.

Various organic and inorganic bases were tested as substitutes for KOAc to get the product in higher yield. Among them, K_2_CO_3_, NaOH, Cs_2_CO_3_ (inorganic bases) and Et_3_N (organic base) in the presence of CH_3_CN resulted in the formation of dihydrothienoindole 80a (Scheme 28), which was finally converted to thieno[2,3-*b*]indole derivative 80 (Scheme 41).

In the synthesis of thieno-[2,3-*b*]indole *via* MBH reaction, firstly proton abstraction at the C-3 position of indoline-2-thione 79 takes place to form an anion, which attacks hydrazinonitroalkene 76*via* Michael addition reaction to form an intermediate that is further activated by the H-bonding property of acetic acid to form 81a. Subsequently, the removal of the nitro group was facilitated by the lone pair of the hydrazine moiety and an acyl iminium-type intermediate 81b was generated. Following this, intramolecular 5-*exo*-trig cyclization of 80 occurred to form dihydrothienoindole 81c, which underwent aerial oxidation to give aromatized thienoindole 80.

**Scheme 42:**
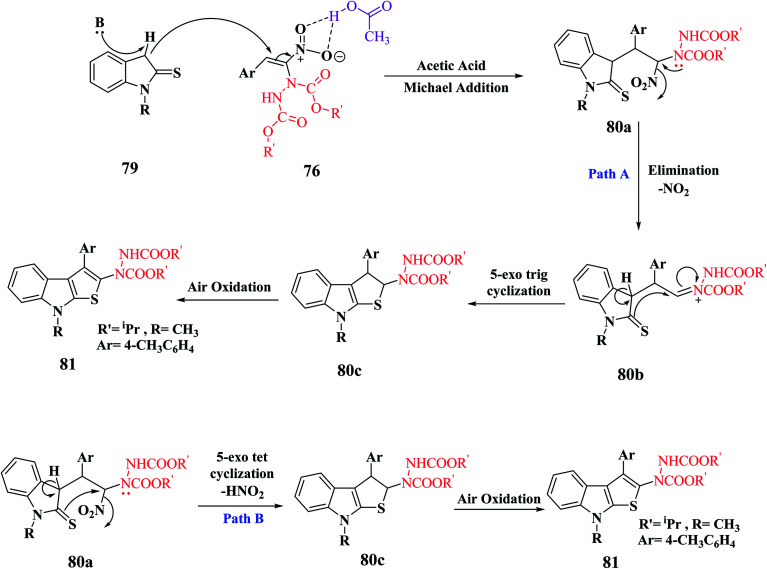
Mechanism for the synthesis of thieno[2,3-*b*]indole.

The reaction can also occur in a different manner, where thio-enolization of 81a takes place initially, and then intramolecular 5-*exo*-tet cyclization and aerial oxidation takes place to form thieno[2,3-*b*]indole 80 (Scheme 42).

**Scheme 43:**
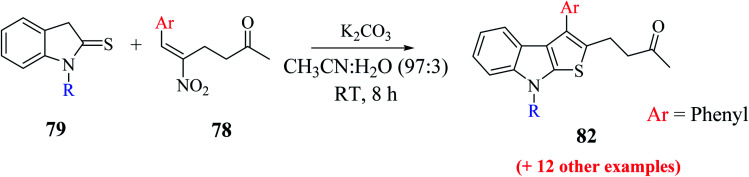
Synthesis of thieno[2,3-*b*]indole *via* RC adduct.

Mane *et al.*^52^ also reported an alternative efficient approach for the synthesis of thieno[2,3-*b*]indole derivatives. They treated substituted RC adduct 78 with N-protected indoline-2-thione 79 in K_2_CO_3_ (1.0 eq.) and CH_3_CN : H_2_O solvent mixture to get 4-(8-methyl-3-phenyl-8*H*-thieno[2,3-*b*]indol-2-yl)butan-2-one 82 in 76% yield (Scheme 43). In addition, it was found that LiCl and H_2_O as additive enhanced the product yield.

**Fig. 8:**
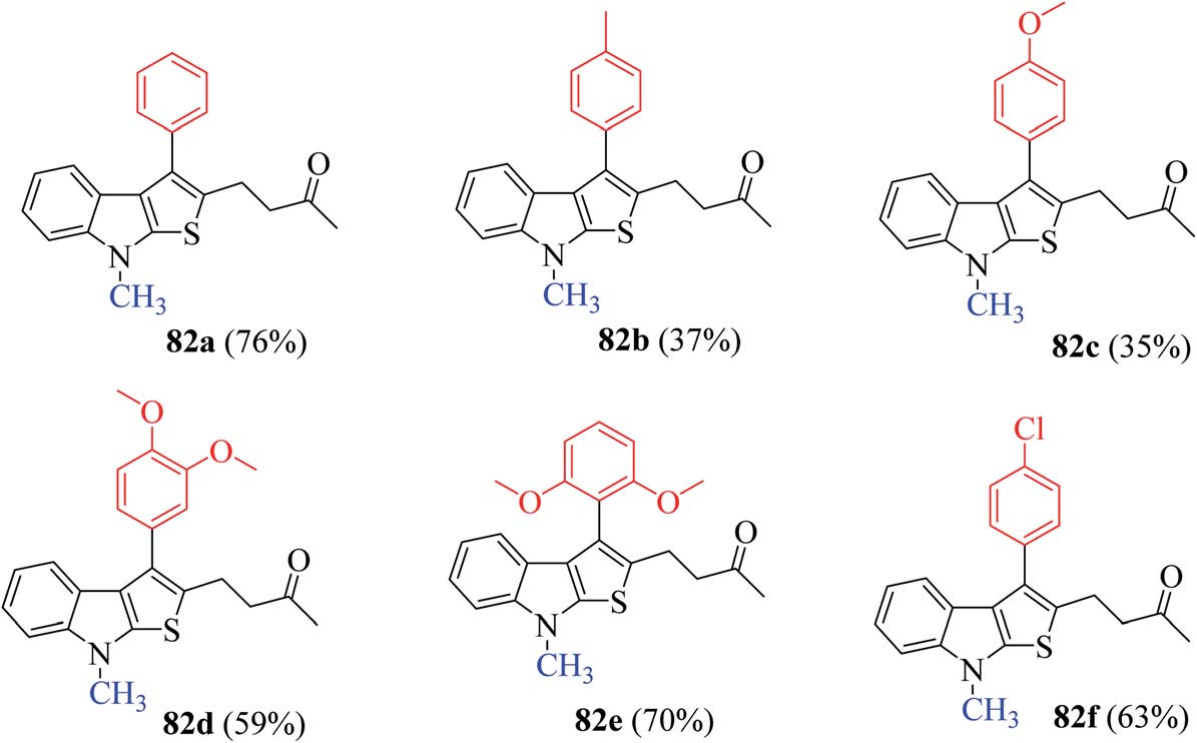
Substrate scope for synthesizing functionalized thieno[2,3-*b*] indoles using several substituted RC-adducts of nitroalkenes.

The presence of electron-donating groups on the aryl ring of RC-adduct 78 significantly reduced the product (82b and 82c) yield with respect to 82a (Scheme 40, Fig. 8).

**Fig. 9:**
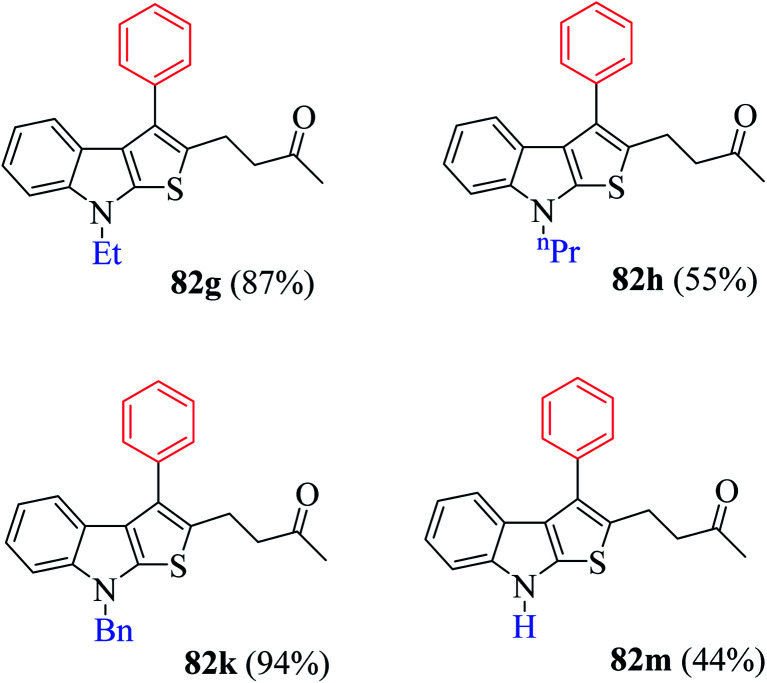
Reaction scope with distinctively substituted indoline-2-thiones.

(1) Presence of numerous electron-donating groups at several positions resulted in good yield of products 82d and 82e.

(2) Presence of weak electron-withdrawing group such as 4-chloro-substituted RC-adduct 82f led to moderate yield (63%) of the product (Fig. 9).

Several N-protecting groups such as ethyl and benzyl resulted in excellent yield of thieno[2,3-*b*]indole derivatives 82g and 82k, whereas groups such as H and *n*-propyl led to a much lower yield of derivatives 82h and 82m (Fig. 9).

**Scheme 44:**
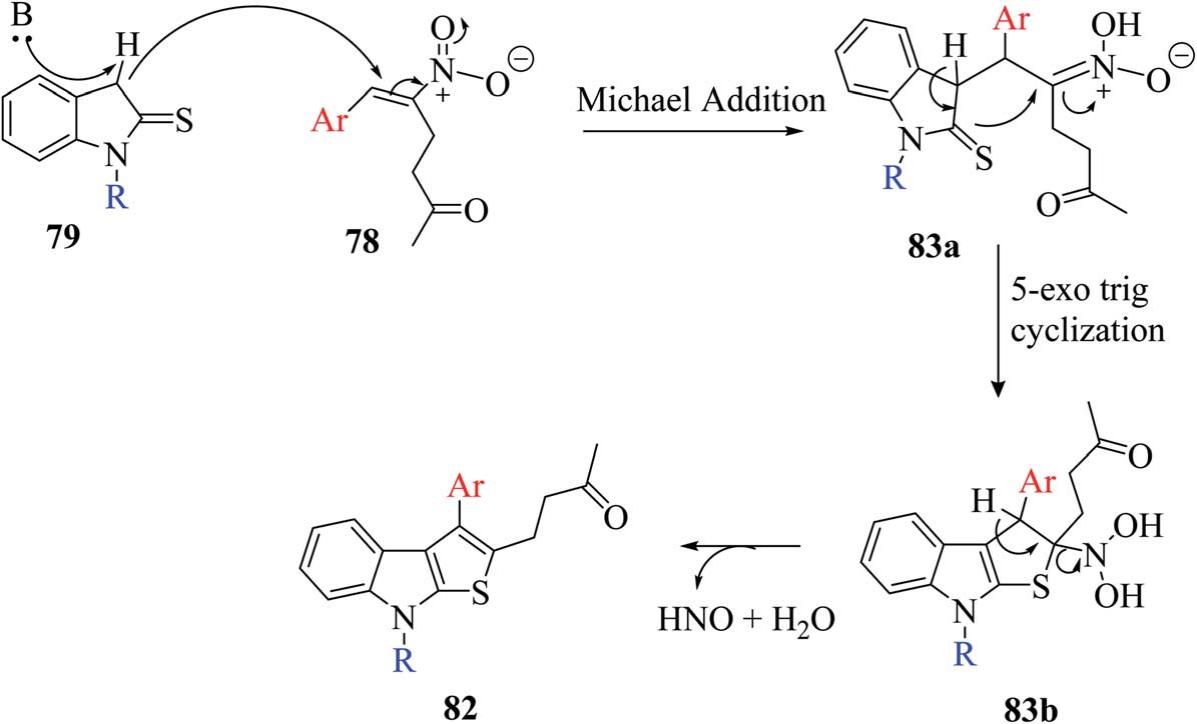
Mechanism for the formation of thieno[2,3-*b*]indole *via* the RC adduct.

The base-directed Michael addition of indoline-2-thione 79 to RC-adduct 78-formed intermediate 83a and intramolecular thio-Mannich-type reaction in 5-*exo*-trig fashion led to intermediate 83b, and further removal of HNO and H_2_O gave the aromatized product. The overall mechanism involved in the synthesis of the thienoindole derivative is depicted in Scheme 44.

### 2.10 Synthesis of thieno[3,2-*b*]indole derivative *via in situ* generation of 3-aminothiophene

It is a convenient method to synthesize thieno[3,2-*b*]indole having a thien-2-yl, aromatic or styryl group at the C-2 position. This method uses 5-substituted-3-aminothiophene-2-carboxylate and proceeds *via* Fischer indolization. It involves two steps, where the first step is the saponification of 3-aminoester with NaOH, and in the second step, this sodium salt reacts with arylhydrazine in glacial CH_3_COOH. In the latter step, decarboxylation of 3-aminothiophene-2-carboxylic acid takes place to give 3-aminothiophene, which further reacts with arylhydrazines under acidic conditions to form arylhydrazone, ultimately undergoing Fischer indolization to give the desired product^58^73.

Thiophene derivatives with an amino group at the C-3 or C-3 and C-4 position have found numerous applications in the formation of thiophene-fused N-heterocycles such as thienoindoles.^59^

#### 2.10.1 Similarity in the behaviour of 3-aminothiophene and thiophene-3(2*H*)-one

**Scheme 45:**
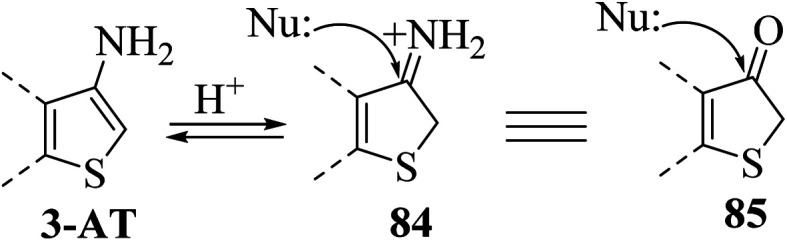
Nucleophilic attack on 3-aminothiophene.

3-Aminothiophene can be considered the synthetic counterpart of thiophene-3(2*H*)-one 85a for annulation reactions. It has been observed that the 3-aminothiophene moiety shows an enamine nature and its protonation takes place on the C-2 position, thereby forming thiophene-3(2*H*)-iminium cation^60^84, which can further react with the nucleophile at the C-3 position to give 85 (Scheme 45).

#### 2.10.2 Strategy for preparing thieno[3,2-*b*]indole derivative

**Scheme 46:**
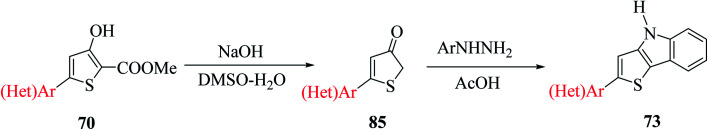
Synthesis of thieno[3,2-*b*]indole from 5(hetero)arylthiophene-3(2*H*)-ones.

Igrashev *et al.* described an efficient method for synthesizing 2-(hetero)aryl-substituted thieno[3,2-*b*]indole 73 by Fischer indolization of arylhydrazines and 5-(hetero)arylthiophene-3(2*H*)-ones 85, which is obtained from 5-(hetero)aryl-3-hydroxythiophene-2-carboxylates 70 (ref. 45) (Scheme 46).

**Scheme 47:**
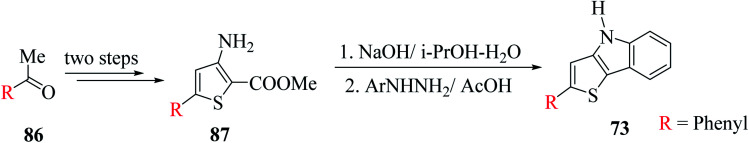
Synthesis of thieno[3,2-*b*]indole from 5-substituted-methyl-3-aminothiophene-2-carboxylates.

Later, they described another method for the synthesis of 2-substituted thieno[3,2-*b*]indoles starting from 5-substituted-methyl-3-aminothiophene-2-carboxylates 87*via* the *in situ* generation of 3-aminothiophene, which further participates in Fischer indolization with arylhydrazines (Scheme 47).

**Scheme 48:**
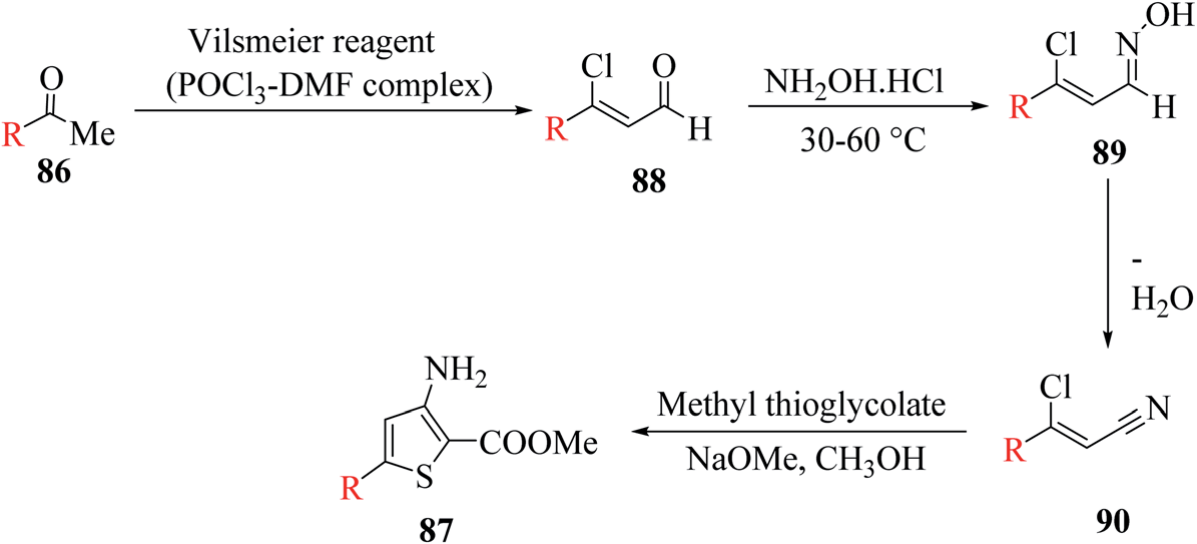
Formation of 5-substituted-3-aminothiophene-2-methylcarboxylate from methyl ketone.

3-Aminothiophene-2-carboxylate 87 was synthesized in two steps. Initially, methyl ketone 86 (R = aryl, styryl or thien-2-yl) was treated with Vilsmeier reagent (POCl_3_–DMF complex) and NH_2_OH·HCl at 30–60 °C to give 3-substituted-3-chloroacrylonitrile,^61–64^ which then reacted with methyl thioglycolate in NaOMe in CH_3_OH solution^65^ to form the product following Fiesselmann method for making the thiophene ring^66^ (Scheme 48).

In the earlier method for the preparation of thieno[3,2-*b*]indole, DMSO was used for the saponification of 3-hydroxythiophene-2-carboxylate. However, the problem was the high temperature required for the reaction due to the initial occurrence of sodium-2-(methoxycarbonyl)thiophen-3-olate. Therefore, the direct conversion of 3-aminothiophene-2-carboxylate was carried out using NaOH in aqueous i-PrOH, without the isolation of the intermediate formed, 3-aminothiophene.

**Scheme 49:**
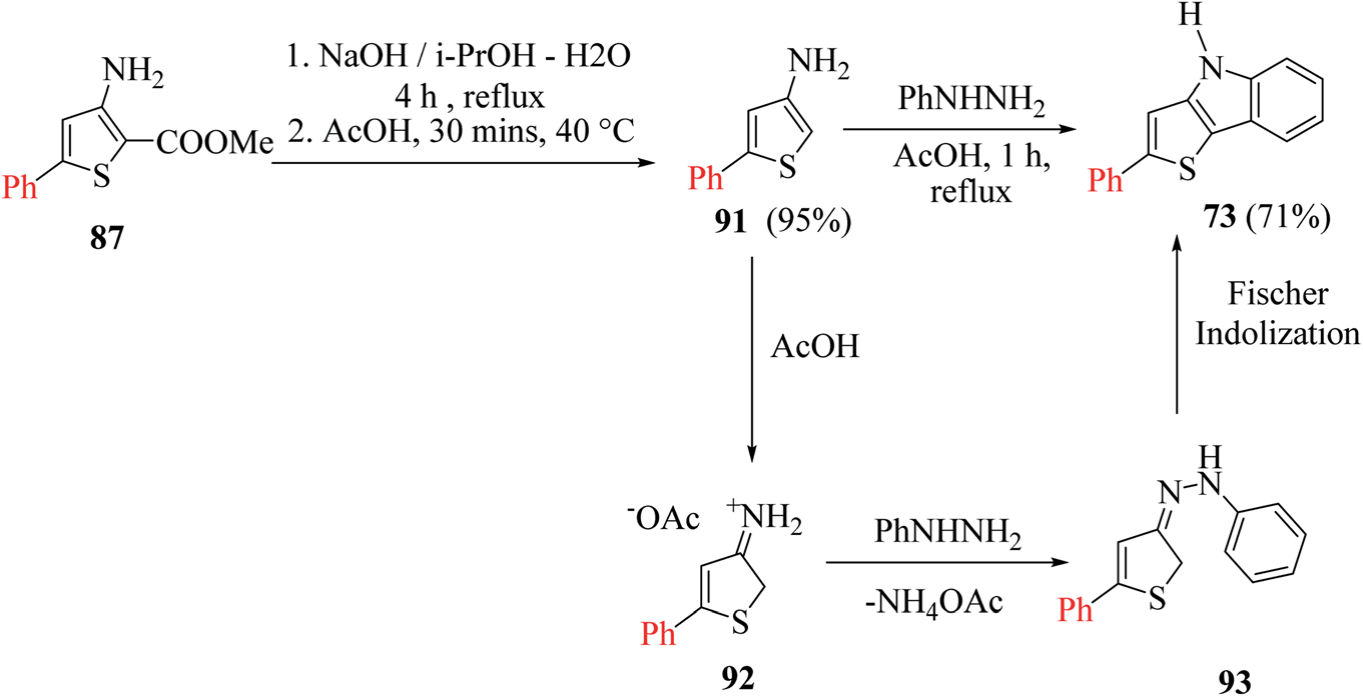
Synthesis of 2-phenylthieno[3,2-*b*]indole.

When 3-aminothiophene-2-carboxylates were substituted at the C-5 position by either aromatic or thien-2-yl groups, 55–77% of 2-substituted thieno[3,2-*b*]indole 73 was obtained (Scheme 49).

### 2.11 Metal-free synthesis of thieno[2,3-*b*]indoles using elemental sulfur

Thieno[2,3-*b*]indoles have been synthesized *via* a base-assisted metal-free approach using cheap and readily available elemental sulfur.^67^ The development of attractive and valuable routes for forming carbonyl group-containing thieno[2,3-*b*]indoles represents several challenges. Here, β-indolyl ketone derivatives were used as effective and easily accessible substrates for the synthesis of carbonyl group-containing thieno[2,3-*b*]indoles through a C=S bond formation reaction.

**Scheme 50:**
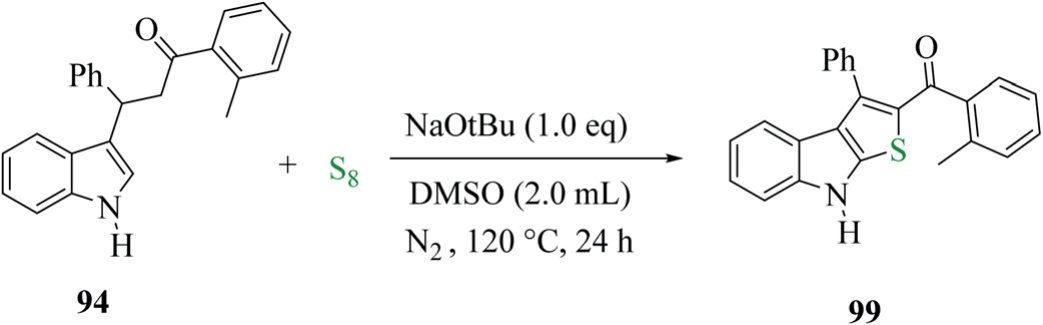
Strategy for preparing 2-substituted-3-phenyl-8*H*-thieno[2,3-*b*]indole 99.

3-(1*H*-indol-3-yl)-3-phenyl-1-(*o*-tolyl)-propan-1-one 94 treated with elemental sulfur and base NaO*t*Bu using anhydrous DMSO as the solvent at 120 °C under N_2_ for 24 h gave product 99 in 97% yield. Moreover, other organic bases, *e.g.*, DBU, gave the product in a minute amount, whereas DABCO yielded the final product in 90%. The quantity of elemental sulfur did not affect the yield of the product^68^ (Scheme 50).

**Scheme 51:**
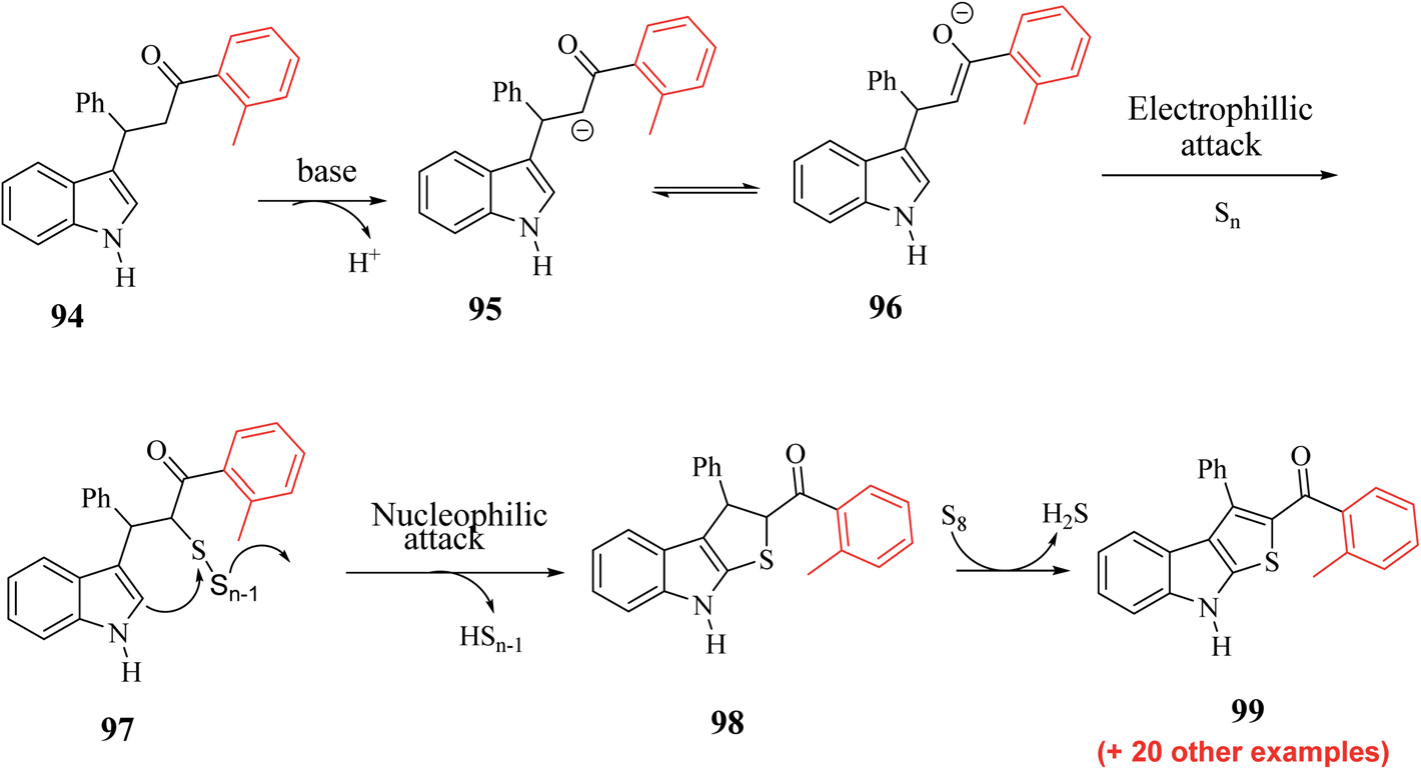
Plausible mechanism for the formation of the product.

Substrate 3-(1*H*-indol-3-yl)-3-phenyl-1-(*o*-tolyl)-propan-1-one 94 was converted to intermediate 95 and remained in proper tautomeric equilibrium with 96. Further, the base abstracted the proton and carbon at the α-position of the CO group was electrophilically attacked by the elemental sulfur, giving intermediate 97. Later, intramolecular nucleophilic cyclization of 97 gave 98 by eliminating elemental sulfur (S_*n*−1_) and oxidative aromatization of elemental sulfur formed 2-substituted-3-phenyl-8*H*-thieno[2,3-*b*]indole 99 (Scheme 51). Hence, a novel synthetic route to poly-substituted thienoindoles was achieved, where S_8_ promoted C=S bond formation to form the desired product.

**Fig. 10:**
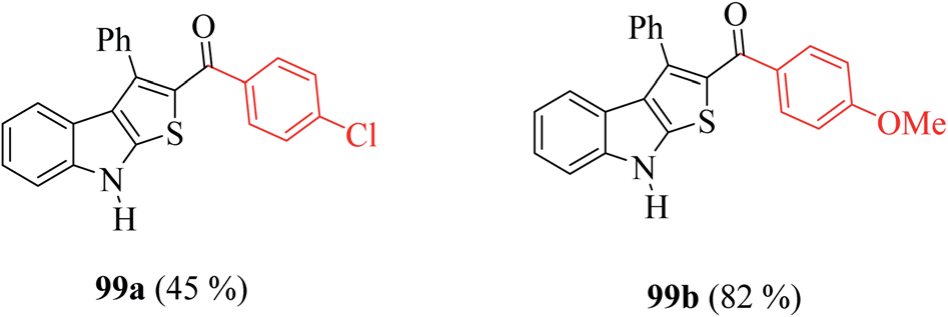
Analogs of thieno[2,3-*b*]indole.

Moreover, the yield of the product decreased when an electron-withdrawing group was substituted at the *p*-position of an extra benzene ring (99a, Fig. 10) and increased in the case of an electron-donating group (99b, Fig. 10).

**Fig. 11:**
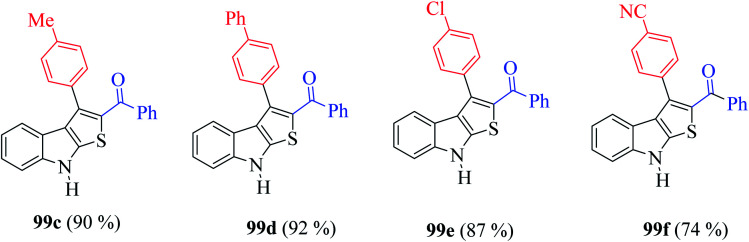
Derivatives of thieno[2,3-*b*]indole.

Furthermore, the presence of methyl, chloride, phenyl and nitrile groups at the *p*-position of the aromatic ring substituted at the C-3 position of the thienoindole derivative resulted in a high yield of the corresponding derivative products 99c–99f (Fig. 11).

### 2.12 Synthesis of thieno[2,3-*b*]indoles *via* green approach using magnetic nanoparticle-supported [urea]_4_[ZnCl_2_] deep eutectic solvent (DES@MNP)

Nguyen *et al.*^69^ reported a green approach for the one-pot three-component synthesis of functionalized thieno[2,3-*b*]indoles *via* the use of a magnetic nanoparticle-supported [urea]_4_[ZnCl_2_] deep eutectic solvent.

**Scheme 52:**
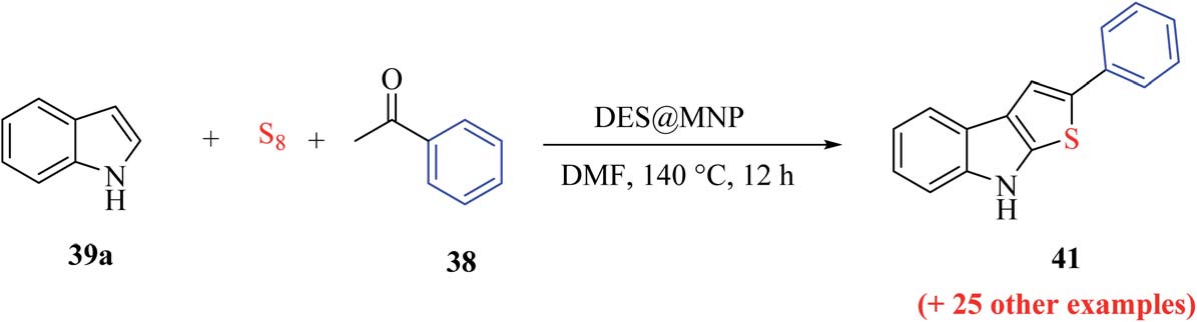
Synthesis of 2-phenylthieno[2,3-*b*]indole 41 from DES@MNP.

The reaction was conducted in the presence of economical and widely accessible reagents, *i.e.*, sulfur, acetophenone 38 and indole 39a, using the magnetic recyclable nanoparticle DES@MNP catalyst in *N*,*N*-dimethylformamide at 140 °C for 12 h. The deep eutectic solvent was prepared with 1.2 g urea in 0.68 g zinc chloride at 100 °C and further mixed with silica-coated Fe_3_O_4_ nanoparticles at 100 °C for 18 h and dried under low pressure at 60 °C for 6 h to form the DES@MNP catalyst. The deep eutectic solvent-covered nanoparticles were beneficial for easy handling, separation and recycling. Moreover, the magnetic nanoparticles have the advantages of easy preparation, high stability, low cost, availability, high surface area and easy separation by a magnet for reuse (Scheme 52).

**Scheme 53:**
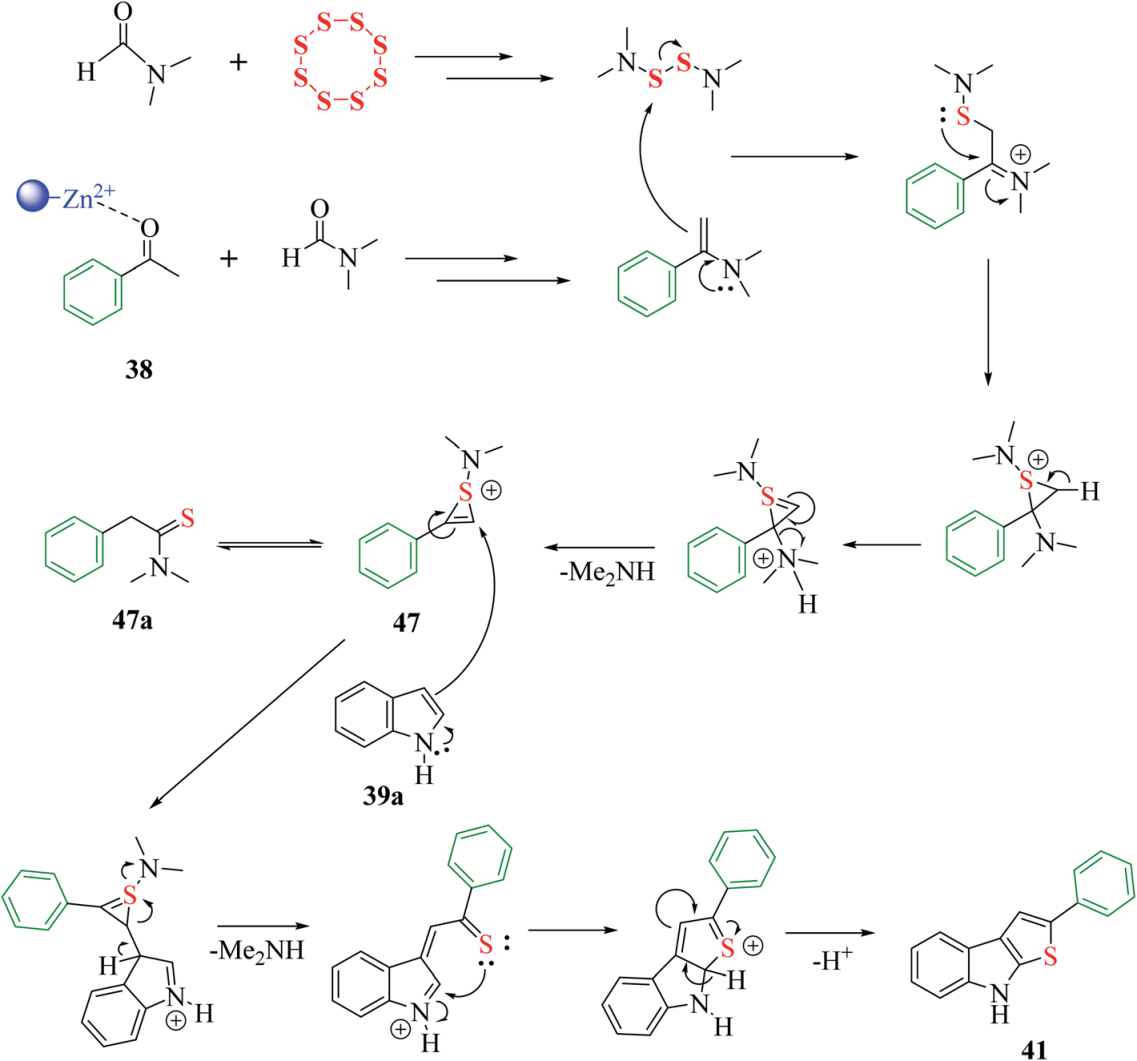
Mechanism for the synthesis of thieno[2,3-*b*]indole *via* nanoparticles.

The proposed mechanism for the synthesis of thieno[2,3-*b*]indole from acetophenone 38, *N*,*N*-dimethylformamide and sulfur aided by the DES@MNP catalyst suggests that the reaction takes place through the formation of 1-(dimethylamino)-2-phenyl-1*H*-thiiren-1-ium 47, which further forms 47a (65%). The resulting intermediate 47 further reacts with indole 39a*via* a ring-opening addition mechanism, ring closure and elimination of dimethyl-amine to form 47 in 87% yield. Furthermore, indole reacts with the reaction mixture, forming 87% product. Moreover, the proposed research led to an efficient protocol having merits, which include simple and efficient recyclable heterogeneous catalyst, vast substrate scope and regioselective product in high yield (Scheme 53).

## 3 Applications of thienoindoles

### 3.1 Biological activities of thienoindoles

**Fig. 12:**
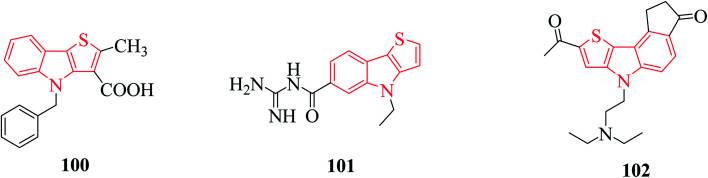
Examples of thienoindole analogs having biological activity.

Thienoindole derivatives show a broad spectrum of biological activities such as anti-bacterial, anti-inflammatory, anti-allergic, anti-viral, anti-tuberculosis activities 100, 5-HT5A receptor binding inhibition 101, antitumor and anti-infective activities 102 (Fig. 12).

**Fig. 13:**
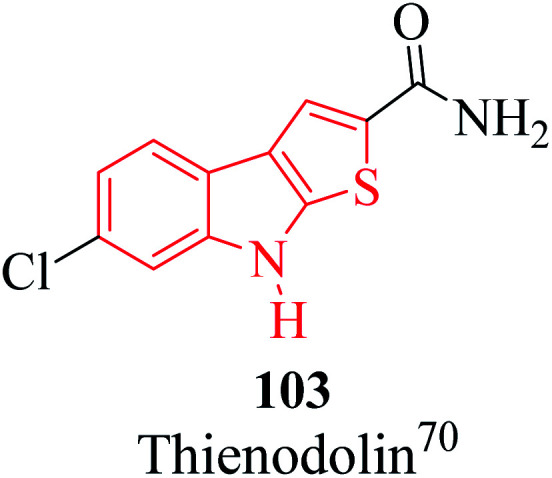
Thienodolin 103, a natural derivative of thieno[2,3-*b*]indole.

The alkaloid thienodolin^70^103 is a natural derivative of thieno[2,3-*b*]indole obtained by the fermentation mixture of *Streptomyces albogriseolus* (Fig. 13). Kanbe *et al.* characterized its activity for plant growth regulation. Furthermore, some thienoindoles are used to treat diseases of the central nervous system and some are potential inhibitors of acetylcholine esterase and butyrylcholine esterase.

### 3.2 Chemical activities of thienoindoles

**Fig. 14:**
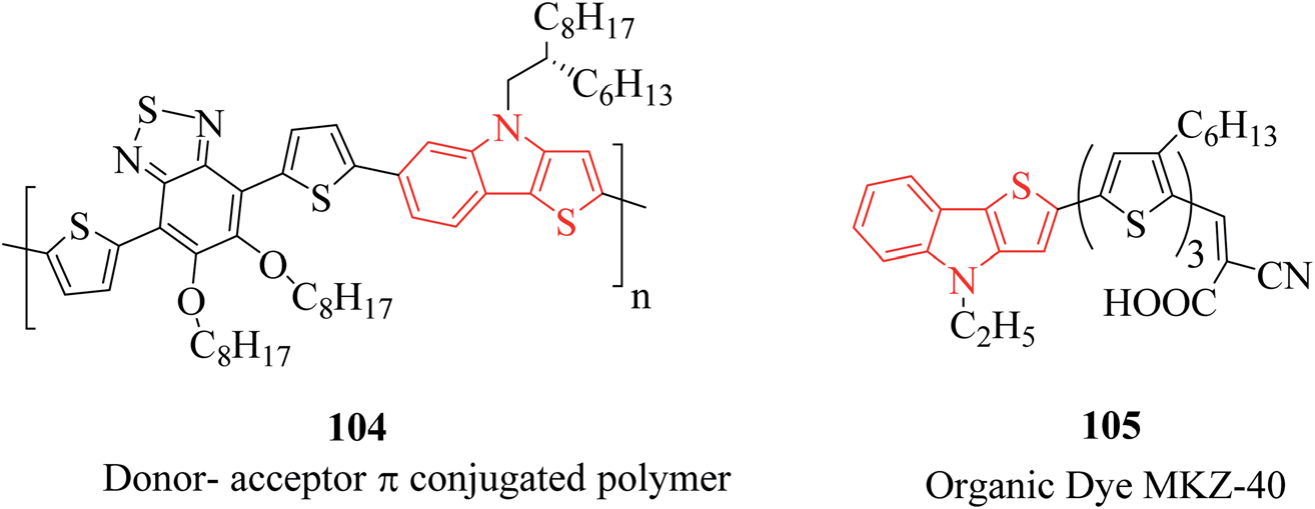
Thienoindole analogs having chemical activities.

Besides their therapeutic properties, thienoindoles are also used for designing molecules of photosensitive and photovoltaic devices because of their π-extended conjugation from electron-rich systems. They are reported to be effective photosensitizers for photothermal and photodynamic therapies and polymers such as PTTICN, PTTIF, and PTTI. Moreover, TI-DTBT3 104 is a donor–acceptor π-conjugated polymer with high charge carrier mobility.^71^ This moiety is interestingly available in several organic dyes such as MKZ-40 105 and DPP-r-TI. They are also used as precursors of polymers used in solar cell applications (Fig. 14).

### 3.3 Electrical activities of thienoindoles

**Fig. 15:**
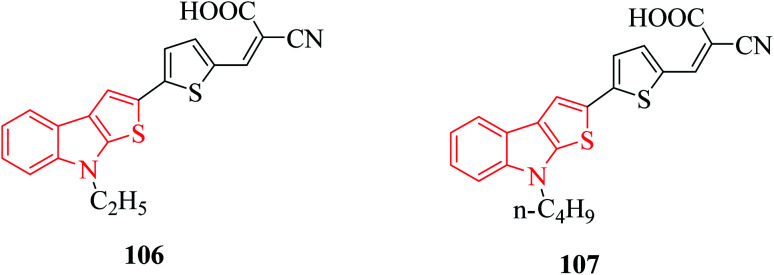
Thienoindole-based compounds having electrical activities in push–pull dyes.

Derivatives of thieno[2,3-*b*]indole are used to design photo- and electroactive compounds, which have been recently assessed in dye-sensitized solar cells (DSSCs). Push–pull dyes IK-1,2 *viz.*, 106 and 107 have been recently reported to be synthesized as a donor part of DSSCs^72^ (Fig. 15).

**Scheme 54:**
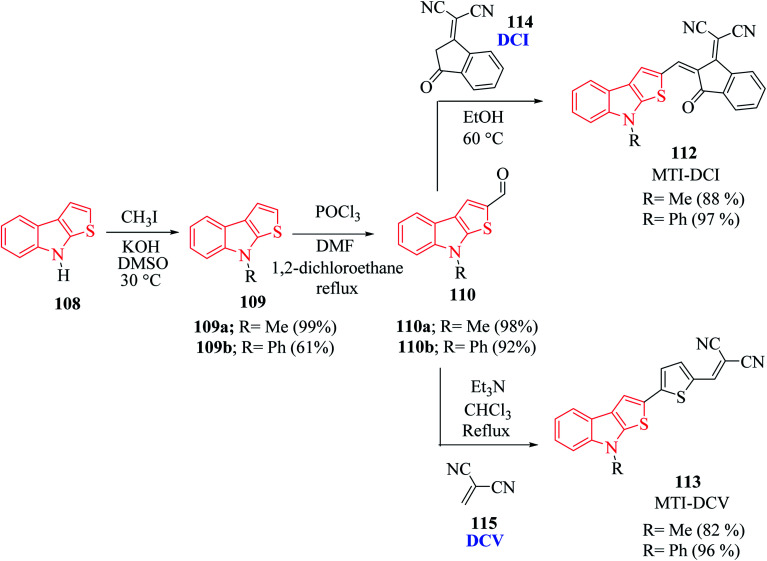
Synthetic approach of push–pull molecules having thieno[2,3-*b*]indole.

**Fig. 16:**
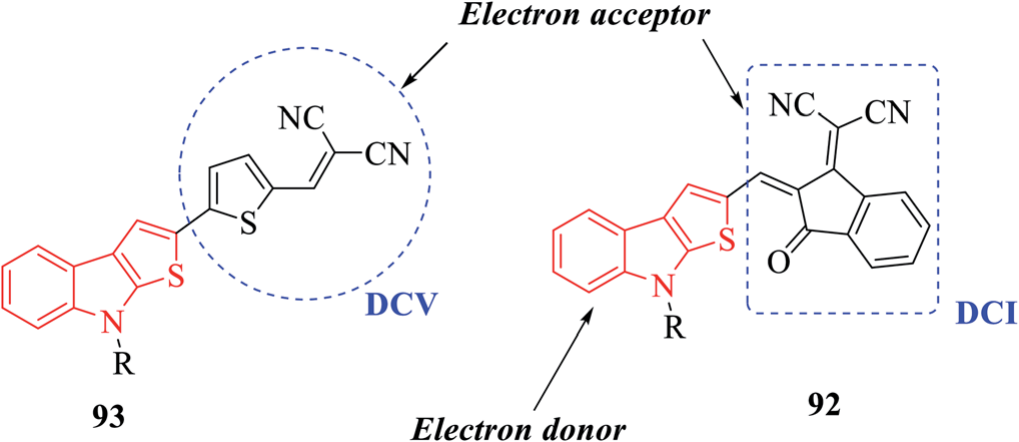
Diagram showing electron-donating and electron-withdrawing parts of push–pull D–π–A molecules.

*N*-Methyl 109a- or *N*-phenyl 109b-substituted thieno[2,3-*b*]indoles are used as the electron donor part of push–pull D–π–A molecules, whereas 2,2-dicyanovinylmethyl (DCV) 115 and (1-(dicyanomethylene)-3-oxo-1-inden-2-ylidene)-methyl (DCI) 114 are used as the electron acceptor^73^ (Scheme 54 and Fig. 16).

MTI-DCV 113 is the smallest push–pull molecule of the series. It has a high absorption coefficient, high thermal stability, good hole transport properties and good absorption in the visible region, and because of all these properties, a bilayer solar cell mostly composed of MTI-DCV showed a power conversion efficiency of more than 1%.

## 4 Conclusions

Thieno[2,3-*b*]indole and thieno[3,2-*b*]indole molecules have been extensively studied because of their wide range of acceptable biological and pharmaceutical applications and other characteristic uses such as in photothermal and organic photovoltaic cells (OPV), making them valuable heterocycles in synthetic organic chemistry, materials chemistry and chemical engineering. Over time, researchers have overcome multiple problems associated with their synthesis such as tedious product separation, problematic catalyst recovery and need for large stoichiometric amounts of solvent. Moreover, the use of highly volatile, toxic and explosive substrates, solvents, and additives for the preparation of thiophene-fused indoles limit their widespread application. Some synthetic protocols also use toxic and corrosive bases such as DABCO, DBU, and LDA and hazardous solvents such as 1,4-dioxane and DMF for the synthesis of thienoindoles. Moreover, a few of the reported methods for their synthesis require the use of functionalized furans, indoles, thiophenes, *etc.* as precursors, which are synthesized *via* multiple steps, making them inefficient. Thus, to overcome the aforementioned issues, newer and convenient synthetic strategies need to be developed, which involve environment-friendly solvents such as water, ethanol, and PEG and reduction in the use of workup solvents. Further, the electronic push–pull mechanism and extended conjugation indicate that thieno[2,3-*b*]indole-based polymers and dyes that display a large variation in properties are still to be discovered. Finally, the future prospects in the arena of thieno[2,3-*b*]indoles synthesis depends on the progress of competent synthetic procedures that can solve the above-mentioned concerns, while keeping environmental-friendliness a priority.

## Conflicts of interest

There are no conflicts to declare.

## Supplementary Material
